# A state wise analysis of the socioeconomic and health impacts of the COVID-19 pandemic in India: lessons for future health system preparedness

**DOI:** 10.3389/fpubh.2026.1710029

**Published:** 2026-07-21

**Authors:** Geetha R. Menon, U. Venkatesh, Jeetendra Yadav, Krushna Chandra Sahoo, Tanu Anand, Aparna Mukherjee, Gunjan Kumar, Alka Turuk, Ashoo Grover, Saurabh Sharma, Sandhya Singh, Firoz Khan

**Affiliations:** 1 Indian Council of Medical Research, New Delhi, India; 2 Department of Community Medicine & Family Medicine, All India Institute of Medical Sciences, Gorakhpur, Uttar Pradesh, India; 3 ICMR-National Institute for Research in Digital Health and Data Science, (Formerly, ICMR-National Institute of Medical Statistics), Medical Enclave Ansari Nagar, New Delhi, India; 4 Health Technology Assessment in India (HTAIn), Department of Health Research, Ministry of Health & Family Welfare, Govt. of India, New Delhi, India; 5 Division of Delivery, ICMR, New Delhi, India; 6 Clinical Studies & Trials Unit (CSTU), Division of Development Research, ICMR, New Delhi, India; 7 Social Statistics Division, National Statistical Office, Ministry of Statistics and PI, Govt of India, New Delhi, India

**Keywords:** cost of productivity life, COVID-19, disability-adjusted life years, GDP, years of potential productivity life lost

## Abstract

**Background:**

The COVID-19 pandemic significantly affected individuals, society, and the national economy. However, there is limited information on the socioeconomic dimension of COVID-19-related health impacts from nationally representative large datasets using an integrated approach. Such information is crucial for tailored policy responses and national preparedness planning. Our study aimed to assess the state-level health and economic impacts of COVID-19 in India.

**Methods:**

The COVID-19 data on age and gender distribution were collated from the Registrar General of India, state dashboards, and the National COVID Clinical Registry. The working population information was obtained from the Periodic Labour Force Survey. We calculated age and gender wise discounted Disability Adjusted Life Years for each state by combining population projections with recovery time and severity percentages. The cost of productivity loss for the working-age population were calculated using each state’s per capita income, to compare the economic impact of COVID-19 and productivity loss disparities across states.

**Findings:**

From March 2020 to September 2021, India recorded 33.6 million COVID-19 cases and 450 thousand deaths with higher proportion of male (65%), indicating a gender disparity in COVID-19 susceptibility. Simultaneously, India incurred a loss of 195.05 billion INR due to mortality and 268.13 billion INR due to absenteeism from work during this period.

**Interpretation:**

The pandemic had serious economic consequences, particularly for the working-age population, resulting in lost productivity from illness or death. To combat future pandemics and reduce the spread of infections and their socioeconomic consequences, national preparedness planning is critical, which includes integrating available nationally representative datasets.

## Introduction

The coronavirus disease (COVID-19) discovered in Wuhan, China in November 2019 (1) has since spread to 238 countries and territories globally. As of January 2025, 777.3 million people have been infected, resulting in 7.08 million deaths (2). The pandemic’s impact has varied across age groups and demographics, with older adults facing higher mortality rate (3) and men experiencing a greater risk of severe illness and death (4). India has recorded the highest number of confirmed cases and deaths in Asia, although the spread has been uneven across the country, with certain states enduring multiple waves of infections. In India, in the first wave approximately 60% of COVID-19 cases occurred among individuals under 45, with a fatality rate of 12% (5). Although a larger share of COVID-19 cases in India during the early phase of the pandemic was observed among younger individuals, mortality was disproportionately concentrated among older age groups, with around 60% or more of deaths occurring in those aged 60 years and above and nearly 85–87% among individuals aged 45 and above. The virus has also significantly impacted younger populations and individuals without pre-existing health conditions, contributing to increased hospitalizations and fatalities.

Accurate data collection is essential for policymakers to effectively allocate resources and mitigate the health and socio-economic consequences of the pandemic. The burden of disease analysis is a valuable tool for measuring the health impact of diseases and associated risk factors. Disability-adjusted life years (DALYs), which combine mortality and morbidity data, serve as a key metric in evaluating the health impact. Estimating COVID-19’s impact using DALYs and Years of Potential Productive Life Lost (YPPLL) can help generate insights across different regions. However, acquiring the necessary data for these estimates poses challenges, as it requires the integration of various data sources.

However, aggregate epidemiological indicators alone do not adequately capture the full health and societal burden of the pandemic. Composite metrics such as disability-adjusted life years (DALYs), which integrate both mortality and morbidity, provide a more comprehensive assessment of disease impact and enable meaningful comparisons across populations and settings.

The COVID-19 pandemic was one of the most critical challenges, with significant consequences for individuals, society, and the national economy. While individuals suffered from health issues and loss of productivity, the national economy experienced reduced output and higher unemployment rates. Numerous studies have explored the health and socioeconomic impact of COVID-19 in India, though most rely on small or localized datasets. These studies, however, have predominantly focused on localized populations, specific clinical outcomes, or short-term impacts, and often lack integration of both health and economic burden within a unified analytical framework. Moreover, few studies have applied standardized burden of disease methodologies to generate comparable state-level estimates across India. However, no studies have provided a comprehensive socioeconomic analysis of COVID-19’s health effects using nationally representative datasets. As a result, there remains a critical gap in nationally representative analyses that simultaneously quantify mortality, morbidity, and productivity losses using standardized metrics such as DALYs and Years of Potential Productive Life Lost (YPPLL).

This study seeks to address that research gap by analysing nationally representative datasets along with morbidity and mortality data from public sources, using a burden of disease approach to assess the health and economic impacts of COVID-19 in India, disaggregated at the state level. By adopting a standardized burden of disease framework and integrating multiple data sources, the study aims to generate policy-relevant, comparable estimates of COVID-19 burden across states, thereby supporting evidence-based decision-making and targeted resource allocation. A substantial socio-demographic and economic variation across states exist in India, which rationales the current study to contextualize the state-level variations in these impacts. The findings of the current study will be helpful to shape the need based resource allocation and preparedness strategies, ultimately contributing to the development of a more resilient healthcare system.

## Methods

### Data sources and triangulation

The study period spans from March 2020 to September 2021, covering the first and second waves of the COVID-19 pandemic in India. The analysis utilized multiple data sources. District wise age-sex distributions of COVID-19 cases were derived from the national COVID-19 testing database (6) maintained by the Indian Council of Medical Research (ICMR). National and state-level projected population data (7) along with COVID mortality figures, were sourced from official counts provided by PRS Legislative Research (8), a trusted digital repository endorsed by the Government of India.

Additionally, the study incorporated state-specific data obtained either directly or through designated health portals like the Kerala COVID-19 Death Information System (CDIS) (9), West Bengal Health Portal (10), Tamil Nadu’s Stop CORONA portal (11) and Karnataka’s COVID Information (12) For states like Maharashtra, Rajasthan, Gujarat, Andhra Pradesh, Odisha, Punjab, Bihar, Jharkhand, Himachal Pradesh, Haryana, Delhi and Chhattisgarh-where age-sex distribution of COVID-19 deaths was not publicly available—the distribution from a published study (13) was used, as these states accounted for approximately 60% of all COVID deaths in India.

The age-sex distribution of COVID cases and deaths were applied from this study to the total counts of infection and deaths obtained from Central and State government websites. While this imputation introduces uncertainty, it enabled inclusion of states accounting for a substantial proportion of national mortality. The implications of this assumption are acknowledged in the limitations, and results should be interpreted with caution. For the north-eastern states where age- and sex-disaggregated mortality data were unavailable, Assam’s age-sex distribution was used as a proxy due to its relatively better data availability and completeness within the region. While acknowledging heterogeneity in demographic composition, healthcare access, and epidemiological patterns across north-eastern states, this approach was adopted to enable inclusion of these states in the national analysis. Given that the combined contribution of these states to total COVID-19 cases and deaths was relatively small, the potential impact of this assumption on overall estimates is limited. However, this approximation may not fully capture state-specific variations and is acknowledged as a limitation. In cases where there was s potential underreporting of State websites, adjustments were made to align with the Ministry of Health and Family Welfare’s counts, ensuring reliable estimates.

Data on the working-age population (15–60 years) was drawn from the 2019–20 Periodic Labour Force survey (PLFS) by Ministry of Statistics and Planning Implementation (MoSPI). The use of PLFS 2019–20 (14) data reflects the most recent nationally representative dataset available for estimating workforce participation and employment structure at the state level. However, it is acknowledged that the COVID-19 pandemic led to substantial disruptions in labour markets, including changes in employment status, migration patterns, and workforce participation, particularly during and after nationwide lockdowns. Due to the absence of comparable, high-quality, and disaggregated labour force data for the pandemic period, the PLFS dataset was used as a baseline approximation. Consequently, the productivity loss estimates presented in this study may not fully capture pandemic-induced labour market shifts and should be interpreted as conservative estimates. State-wise age and sex-specific life expectancies for 2020 were obtained from Registrar General of India (RGI) website (15). The study also utilized data on the proportion of cases at various disease severity levels from the National Clinical Registry for COVID-19 (NCRC) (16), providing crucial insights into the progression of COVID-19 and its implications for healthcare resource management. The NCRC was real-time data portal initiated by Indian Council of Medical Research in collaboration with the Ministry of Health and Family Welfare, Government of India for recording data on COVID-19 related illness of 51,781 hospitalized patients across a network of 42 hospitals across the country during 2020–24.

### The estimation method

The clinical progression of COVID-19 from infection to recovery or death has been adapted from the model developed by McArthur et al. (17). Due to the absence of data on post-recovery or hospital discharge, this analysis does not account for the burden associated with post-COVID-19 complications or long-term functional impairments.

### Health burden indices

The Burden of Disease was calculated following the protocol for country studies developed by the European Burden of Disease Network (18). The health burden indices were estimated using the standard formula for Disability Adjusted Life Years (DALYs); DALYs = YLD + YLL where YLD represent the years lived with disability, reflecting the number of years an individual spends in ill health and YLL refers to the years of life lost due to premature death, defined as dying before the ideal life expectancy.

While the Global Burden of Disease study (19) has removed the assumptions of time and age preferences in its latest estimates, these factors are still crucial for evaluating the future economic impact. As a result, they have been included in study’s analysis.

The formula for calculating Years of Life Lost (YLL) with uniform age weighting and a 3% discount rate is (20).

YLL_discounted_ = $\frac{1-e^{-\mathrm{rLa}}}{r}\;\;$ where *r* = 0.03 is the discount rate and *L_a_* is the standard life expectancy at age *a*.

The formula for calculating Years Lived with Disability (YLD) using the incidence-based approach with uniform age weighting and *r* = 0.03 as the discount rate is

$${\mathrm{YLD}}_{\text{discounted},i,s}=I_{i,s}\times D_{i,s}x\;\frac{1-e^{-rD_{i,s}}}{r}\times D_{\mathrm{Wi},s}$$

where:

- *r* = 0.03 is the discount rate,
- *I*_*i*,*s*_ is the incidence of COVID-19 in the *i*^th^ age group at the *s*^th^ stage/sequelae,
- *D*_*i*,*s*_ is the duration of disability due to COVID-19 in the *i*^th^ age group at the *s*^th^ stage/sequelae.
- *D*_*Wi*,*s*_ is the disability weight for *s*^th^ sequelae of COVID-19 in the *i*^th^ age group, with disability weight ranging from 0 (complete health) to 1 (death).

A 3% discount rate is conventionally used in DALY calculations because DALYs are intended to estimate the overall burden of disease in a standardized and internationally comparable manner, following Global Burden of Disease (GBD) guidelines. In this study, the stages of COVID-19 was defined as per the COVID19 Clinical Management Protocol Algorithm Adults COVID-19 provided by the Ministry of Health and Family Welfare, Government of India (21). The disability weights for the different stages of COVID-19 were adapted from a published study (22) which in turn were derived from the Global Burden of Disease (GBD) 2019 disability weights for lower respiratory tract infectious diseases (23). We acknowledge that these disability weights may not fully reflect context-specific perceptions of disease severity and functional impairment across different sociocultural settings, including India. However, in the absence of India-specific disability weight estimates for COVID-19, published disability weights from the existing literature were used. The duration of disability for patients in home isolation was assumed to be 7 days, even though the Government of India guidelines (24) recommended a 14-day home quarantine. In DALY calculations, disability duration should reflect the time a person is unwell or functionally limited, not just the time they are asked to stay at home. Even though home quarantine for COVID-19 lasted 14 days, many mild cases had symptoms for only about 7 days. The rest of the quarantine period was mainly a precaution to prevent spread, not a period of actual disability. So, we used 7 days as the disability duration to capture the period of real illness for home isolated cases. For hospitalized patients, the duration of disability was determined for each decennial age group using data from the National Clinical Registry for COVID-19 (NCRC). The percentage of asymptomatic varied between the initial stages of Wave 1 and later in wave 2 it declined as per the newspaper reports. Localized studies from India (25, 26) and news reports showed approximately 91% COVID cases were asymptomatic or mild during the first and early second waves and were managed through home isolation as per Government of India guidelines. This proportion would have changed during other waves but due to non-availability of published data, we assumed approximately 91% cases did not require any hospitalization during the study period. The percentage of hospitalized cases at different levels of severity was obtained from the NCRC. These proportions were assumed to be consistent across all states (Table 1).

**Table 1:** COVID-19 disease stages, proxy data inputs, and assigned disability weights.

Stage of the disease	Data input as proxy	Disability weight
Asymptomatic or mild (home isolated)	91% did not require any hospitalization	0.051
Mild/moderate (hospitalized)	Percentage of hospitalized cases classified as mild/moderate from the NCRC	0.051
Severe (hospitalized)	Percentage of hospitalized cases classified as severe from the NCRC	0.133
Critical (hospitalized)	Percentage of hospitalized cases classified as critical from the National COVID Clinical Registry	0.655

For the sake of comparison across countries/regions/states, we have reported age-adjusted YLL, YLD, DALYS, deaths, and infection rates per 100,000 with their 95% confidence interval. These indices were calculated as follows:

$$\mathrm{Age}\;\text{adjusted rate}\;R=\sum w_{i}\times r_{i}$$

$w_{i}$ is the standard population weights = $\frac{N_{i}^{\mathrm{std}}}{\sum N_{i}^{\mathrm{std}}}$

$N_{i}^{\mathrm{std}}$ : world standard population in age group *i*

$r_{i}$ = age specific rate. For example, the age specific YLL rate for the age group *i* = $\frac{{\mathrm{YLL}}_{i}}{n_{i}}$ where *n_i_* is the population at risk. Assuming a Poisson distribution for each age specific rate *r_i_* (especially if rates are above 20) the variance of *R* = $\sum w_{i}^{2}\times \mathrm{Var}(r_{i})$ = $\sum (w_{i}^{2}\times \frac{r_{i}}{n_{i}})$ ,

SE(R) = $\sqrt{\sum (w_{i}^{2}\times \frac{r_{i}}{n_{i}})}$ . 95% Confidence Interval = R ± 1.96·SE(R). For age specific rates below 10, Poisson distribution does not hold good, so we have calculated the 95% CI using bootstrapping.

### Economic burden indices

The cost of lost productivity (CPL) was calculated using the human capital (27), which considers three key parameters: the length of time absent from work due to illness, the market wage, and the labour force participation rate. The human capital approach was selected for this analysis as it provides a comprehensive estimation of productivity losses by valuing the total potential income lost due to absenteeism from work and premature mortality. This approach is particularly relevant in the context of large-scale public health emergencies such as the COVID-19 pandemic, where long-term economic impacts and workforce disruptions are substantial. Alternative approaches, such as the friction cost method, which estimates productivity loss only during the period required to replace a worker, may underestimate the broader societal impact of premature mortality and prolonged illness. Similarly, willingness-to-pay approaches, while capturing individual preferences, require detailed primary data that are not readily available in nationally representative datasets. Given the objectives of this study and the use of secondary national data sources, the human capital approach was considered the most appropriate and policy-relevant method for estimating economic burden.

Years of Potential Productive Life Lost (YPPLL) (28) due to COVID-19 was estimated separately for males and females. The YPPLL was valued using the per capita income for each state as an approximation of the foregone productivity during the period of absence and the years of working life lost due to premature mortality.

The formula for YPPLL is:

$$\text{YPPLL}=\sum_{i=1}^{n}D_{i}\times w_{i}\times \frac{1}{{\left(1+d\right)}^{\mathrm{wi}-1}}\;|\;\;i=1,2,\ldots ,n$$

where:

- *i* represents the *i*th working age group,
- *n* is the age group that ends at 60 years,
- Di is the number of deaths at age *i*;
- *w_i_* is the productive years remaining at age of death (calculated as 60 minus the midpoint of the *i*th age group),
- *d* is the discount rate, which was set to the bank rate proposed by the Reserve Bank of India (RBI) = 0.0425 (29).

For YPPLL calculation a discount rate of 4.25% is used because YPPLL estimates the economic opportunity cost and present valuation of future productive earnings which are discounted using an economic or financial discount rate aligned with the RBI bank rate during the pandemic period. For each state, cost of Productivity loss (CPL) was estimated for both absenteeism (temporary) from work and premature mortality (permanent losses), using the following formulas:

$${\mathrm{CPL}}_{\text{Premature mortality}}=\sum_{i=1}^{n}{\text{YPPLL}}_{i}*\mathrm{per}\;\text{capita}\;\mathrm{GDP}*P_{i}$$

$${\mathrm{CPL}}_{\text{absenteeism from work}}=\sum_{i=1}^{n}S*L_{i}*N_{i}*P_{i}$$

where

- *P_i_* is the proportion of working population, in the *i*^th^ age group, in that state
- *S* is the mean daily salary, calculated as the per capita income (30) of that state by 313 (number of paid working days in a year accounting for 52 Sundays)
- *L_i_* is the mean recovery time for the *i*^th^ age group
- *N_i_* is the number of incident cases in the *i*^th^ age group

The costs were adjusted to reflect 2021 values. Recovery times for patients at different stages of illness were obtained from the National Clinical Registry for COVID-19 (NCRC) and used as a proxy measure for work absenteeism. The average duration of absenteeism was assumed to be 14 days for asymptomatic and mild cases, 21 days for moderate cases, and 30 days for severe cases.

**Figure 1:**
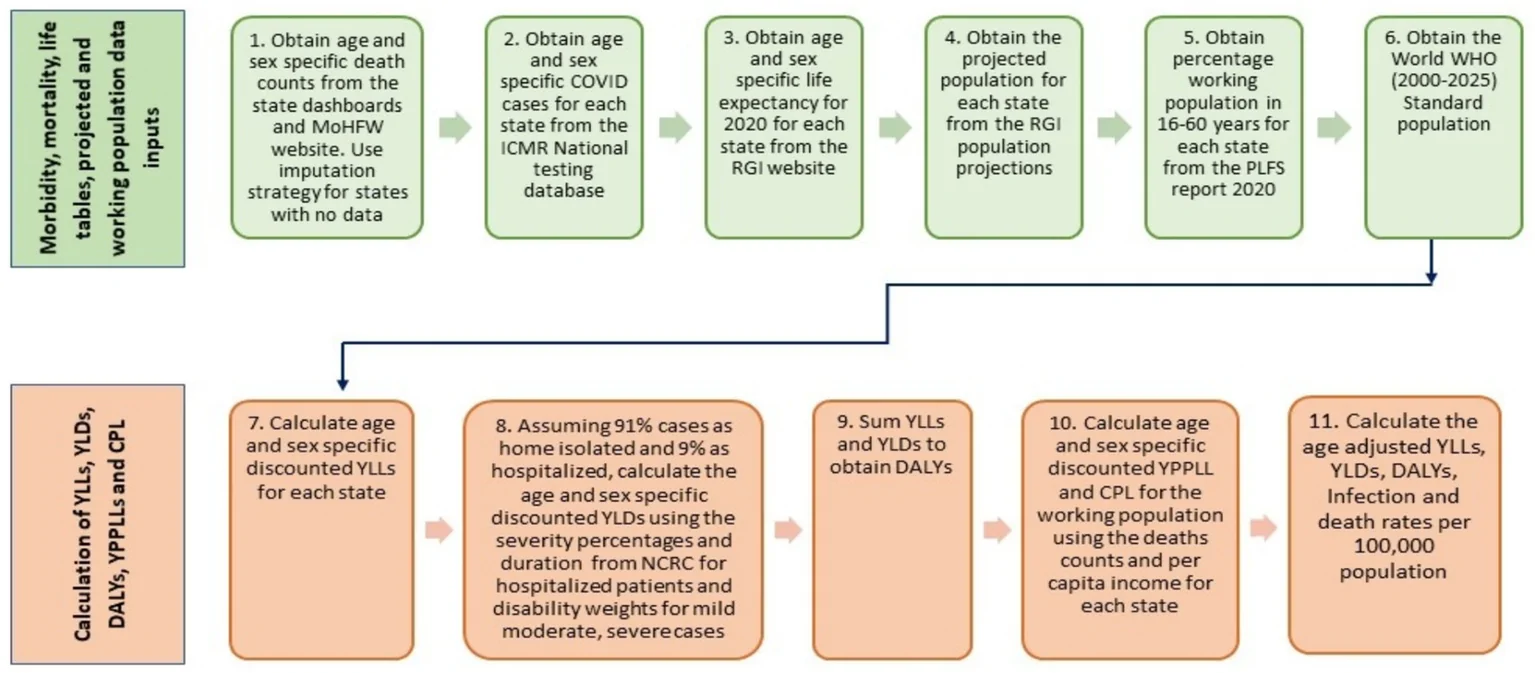
Summary of the steps in the disease and economic burden estimation process. YLD = year lived with disability. YLL = year of life lost. DALY = disability-adjusted life-year. YPPLL = year of potential productive life lost. CPL = cost of productive life. NCRC = National Clinical Registry for COVID 19. RGI = Registrar General of India. MoHFW = Ministry of Health and Family Welfare, Government of India.

The estimations were done on an excel sheet after extracting the relevant morbidity and mortality data from various data sources and organizing them by age and gender in the required format. The step-by-step estimation process is outlined in Figure 1.

## Findings

The proportion of hospitalized patients across the three severity stages and the duration of disability experiences by COVID-19 patients are presented in Table 2. The duration of disability is calculated as the third quartile, measured from the onset of COVID-19 symptoms to the date of hospital discharge. This suggests that older adults, particularly those over 40 experience more severe illness and longer periods of disability.

**Table 2:** Severity percentage among hospitalized cases and average days of recovery.

Age-group	Percentage (%) among hospitalized cases			Average days to recovery/discharge		
	Mild/moderate	Severe	Critical	Mild/moderate	Severe	Critical
<1	70	20	10	11	14	14
1–5	81	14	4	10	13	22
5–10	88	9	3	11	16	14
10–15	87	10	3	11	16	20
15–20	87	12	1	11	13	23
20–25	87	12	1	12	15	21
25–30	81	17	2	12	15	23
30–35	72	25	3	12	16	19
35–40	64	32	4	13	16	21
40–45	58	36	6	13	16	22
45–50	56	40	5	12	17	22
50–55	52	42	6	13	17	22
55–60	52	41	7	13	17	22
60–65	49	44	7	13	17	22
65–70	48	46	6	13	17	24
70–75	47	49	5	13	18	23
75–80	44	51	6	12	18	25
80+	43	52	5	13	18	22

As of September 30, 2021, India recorded approximately 33.6 million confirmed cases of COVID-19, with males accounting for 61% (20.05 million) of the total cases. The state-wise distribution showed Maharashtra as having the highest incidence, with about 6.58 million cases, followed by Kerala (3.41 million), Karnataka (3.24 million), Tamil Nadu (2.8 million), and Andhra Pradesh (2.29 million). India’s cumulative death toll reached 450,000, with Maharashtra, with 139,000 fatalities, emerged as the state with the highest death toll, followed by Karnataka (37,777), Tamil Nadu (35,579), Delhi (25,530), Kerala (24,963), and Uttar Pradesh (22,891). Notably, Himachal Pradesh reported the lowest death count among the larger states (3,653), while the collective total for the seven north-eastern states was the lowest regional death toll (Table 3).

**Table 3:** Distribution of COVID-19 cases and deaths by gender across Indian states.

States	COVID cases			COVID deaths		
	Males	Females	Both	Males	Females	Both
India	19,998,841	13,596,902	33,595,743	295,462	154,575	450,037
Andhra Pradesh	1,320,377	970,651	2,291,028	9,349	4,814	14,163
Assam	375,938	241,172	617,110	4,111	1,762	5,873
Bihar	525,099	233,206	758,305	6,376	3,284	9,660
Chhattisgarh	613,149	416,625	1,029,774	8,953	4,610	13,563
Delhi	899,892	617,580	1,517,472	16,852	8,678	25,530
Goa	106,651	76,617	183,268	2,409	1,244	3,653
Gujarat	583,505	348,491	931,996	6,663	3,431	10,094
Haryana	565,723	354,676	920,399	6,518	3,356	9,874
Himachal Pradesh	122,812	85,667	208,479	2,418	1,245	3,663
Jammu and Kashmir	200,514	108,665	309,179	2,725	1,701	4,426
Jharkhand	259,034	134,857	393,891	3,392	1,746	5,138
Karnataka	1,917,446	1,325,387	3,242,833	24,188	13,589	37,777
Kerala	1,788,680	1,617,560	3,406,240	14,480	10,483	24,963
Madhya Pradesh	598,912	360,081	958,993	7,048	3,472	10,520
Maharashtra	3,923,872	2,657,355	6,581,227	91,759	47,252	139,011
Odisha	565,723	354,676	920,399	5,411	2,787	8,198
Punjab	432,200	258,132	690,332	10,902	5,614	16,516
Rajasthan	760,030	394,589	1,154,619	5,910	3,044	8,954
Tamil Nadu	1,632,873	1,166,368	2,799,241	23,751	11,828	35,579
Telangana	83,901	50,126	134,027	3,499	1,545	5,044
Uttar Pradesh	1,242,043	663,597	1,905,640	15,164	7,727	22,891
Uttarakhand	217,941	127,947	345,888	4,983	2,410	7,393
West Bengal	921,649	683,147	1,604,796	12,601	6,207	18,808
Other Northeast	198,924	248,833	447,757	3,987	1,709	5,696
Other Union territories	141,953	100,897	242,850	2,013	1,037	3,050

In India, COVID-19 cases resulted in a total of 1,182 years with disability, with 62% of this burden borne by males. The pandemic’s initial waves led to an estimated 6.7 million YLLs, with males accounting for 64.3%. Maharashtra had the highest YLL at 2.17 million, constituting 32.3% of the national total (Supplementary Table S14). The age adjusted YLL, YLD, DALYs, deaths and infections rates per 100,000 was estimated to compare the rates across the different states (Table 4). Goa had the highest age adjusted rate (3071.1) for YLLs because of the high death rates per 100,000. Compared to other states, Goa had a very high infection rate per 100,000 and hence the morbidity index (YLD) was comparatively highest 11,194 (197.36, 11,194.00).

**Table 4:** Burden of COVID-19 in India: state-wise adjusted estimates of YLLs, YLDs, DALYs, deaths, and infections.

States	Adj YLL 95% CI	Adj YLD 95% CI	Adj DALYs 95% CI	Adj deaths 95% CI	Adj infections 95% CI
Andhra Pradesh	303.399 (303.395–303.404)	0.135 (0.105–0.165)	303.53 (302.10–304.97)	25.31 (24.89–25.72)	4,113.2 (4,107.87–5,207.24)
Assam	323.686 (323.680–323.693)	0.055 (0.030–0.080)	323.74 (321.80–325.69)	19.04 (18.56–19.53)	1,740.03 (1,735.69–1,768.76)
Bihar	174.788 (174.786–174.792)	0.021 (0.0127–0.0311)	174.81 (173.88–175.74)	13.99 (13.71–14.27)	720.12 (718.49–952.04)
Chattisgarh	715.64 (715.630–715.651)	0.114 (0.0750–0.1533)	715.75 (712.45–719.06)	58.56 (57.57–59.55)	3,522.95 (3,516.14–3,529.75)
Delhi	2,263.124 (2,263.103–2,263.147)	0.243 (0.1765–0.3108)	2,263.37 (2,256.52–2,270.21)	146.17 (144.38–147.97)	7,139.28 (7,127.92–8,553.65)
Goa	3,071.005 (3,070.92–3,071.09)	0.443 (0.44–0.4469)	3,071.45 (3,044.36–3,098.54)	236.48 (228.81–244.15)	11,194 (197.36–11,194.00)
Gujarat	331.738 (331.733–331.74)	0.065 (0.0425–0.0890)	331.8 (330.14–333.47)	22.51 (22.07–22.94)	1,980.85 (1,976.83–1,984.87)
Haryana	573.556 (573.548–573.566)	0.101 (0.0651–0.1386)	573.66 (570.77–576.55)	38.52 (37.76–39.28)	3,093.84 (3,087.52–3,100.16)
Himachal Pradesh	724.668 (724.649–724.687)	0.086 (0.0219–0.1521)	724.76 (718.88–730.63)	45.27 (43.80–46.74)	2,667.48 (2,656.02–2,678.93)
Jammu and Kashmir	588.209 (588.196–588.224)	0.078 (0.0304–0.1268)	588.29 (583.91–592.67)	38.95 (37.80–40.09)	2,233.88 (2,226.00–2,241.75)
Jharkhand	252.143 (252.138–252.149)	0.033 (0.0147–0.0531)	252.18 (250.39–253.96)	18.24 (17.74–18.74)	1,077.88 (1,074.51–1,081.24)
Karnataka	765.059 (765.053–765.066)	0.149 (0.1211–0.1784)	765.21 (763.14–767.28)	56.69 (56.12–57.26)	4,624.62 (4,619.59–4,629.66)
Kerala	687.942 (687.935–0.000)	0.312 (0.2559–0.3691)	688.26 (685.84–690.67)	53.91 (53.25–54.58)	9,415.89 (9,405.01–9,426.78)
Madhya Pradesh	221.107 (221.104–221.111)	0.066 (0.0485–0.0842)	221.17 (220.08–222.26)	15.53 (15.23–15.82)	1,200.06 (1,197.66–1,202.46)
Maharashtra	1,704.331 (1,704.325–1,704.339)	0.174 (0.1517–0.1980)	1,704.51 (1,702.24–1,706.78)	110.66 (110.07–111.24)	5,247.57 (5,243.56–5,251.58)
Odisha	286.166 (286.162–286.172)	0.062 (0.0402–0.0856)	286.23 (284.68–287.78)	18.28 (17.89–18.68)	1,956.65 (1,952.66–1,960.65)
Punjab	799.773 (799.764–799.784)	0.069 (0.0044–0.1355)	799.84 (792.34–807.35)	51.14 (50.36–51.92)	2,137.69 (2,125.51–2,149.8)
Rajasthan	211.014 (211.011–211.018)	0.045 (0.0299–0.0605)	211.06 (209.93–212.19)	14.69 (14.38–14.99)	1,480.72 (1,478.02–1,483.42)
Tamil Nadu	530.031 (20.943–20.943)	0.214 (0.1828–0.2457)	530.25 (528.76–531.73)	38.38 (37.98–38.77)	3,364.92 (3,360.98–3,368.86)
Telangana	205.95 (205.946–205.955)	0.014 (0.0020–0.0263)	205.96 (200.66–207.41)	13.59 (13.21–13.96)	341.13 (339.30–342.96)
Uttar Pradesh	194.286 (194.285–194.289)	0.029 (0.0217–0.0366)	194.32 (193.68–194.95)	13.04 (12.87–13.21)	900.58 (899.30–901.86)
Uttarakhand	1,056.323 (1,056.304–1,056.344)	0.104 (0.0446–0.1644)	1,056.43 (1,050.24–1,062.62)	70.82 (69.20–72.43)	2,979.93 (2,969.99–2,989.86)
West Bengal	248.01 (248.008–248.014)	0.029 (0.0191–0.0398)	248.04 (247.09–248.99)	18.45 (18.19–18.71)	1,517.6 (1,515.25–1,519.94)
Other northeast	680.805 (680.792–680.819)	0.089 (0.0432–0.1359)	680.89 (676.74–685.05)	40.05 (39.01–41.09)	2,736.99 (2,677.83–2,796.15)
Other union territories	2,010.285 (2,010.216–2,010.354)	0.228 (0.0684–0.3885)	2,010.51 (1,992.14–2,028.89)	139.53 (134.58–144.48)	6,341 (6,315.78–6,366.22)

The total DALYs associated with COVID-19 in India amount to 6.70 million, with males comprising 65% of these lost years. Males generally accounted for 62–68% of the national disease burden; however, exceptions were observed in Jammu and Kashmir and Kerala, where DALYs attributed to females were higher, suggesting relatively greater female mortality in these regions (Supplementary material).

**Figure 2:**
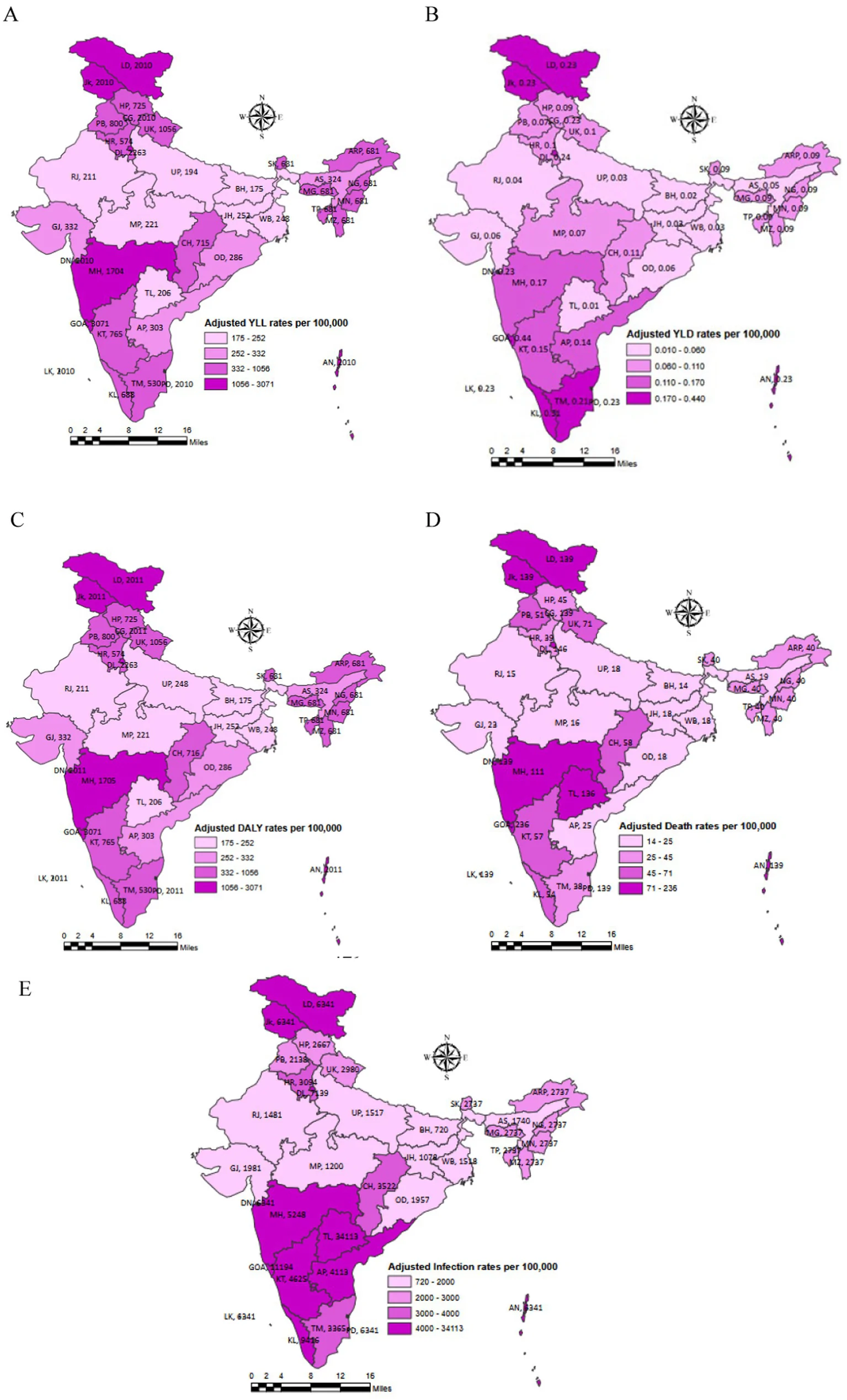
State-wise variation in age-adjusted rates per 100,000 for YLL, YLD, DALYs, infections and deaths. AN = Andaman & Nicobar Islands, ARP = Arunachal Pradesh, BH = Bihar, CG = Chandigarh, DL = Delhi, AS = Assam, CH = Chhattisgarh, DN = Dadra & Nagar Haveli, DD = Daman & Diu, GOA = Goa, GJ = Gujarat, HR = Haryana, HP = Himachal Pradesh, JH = Jharkhand, KT = Karnataka, KL = Kerala, LP = Lakshadweep, MP = Madhya Pradesh, MH = Maharashtra, MN = Manipur, MG = Meghalaya, MR = Mizoram, NG = Nagaland, PD = Puducherry, PB = Punjab, RJ = Rajasthan, SM = Sikkim, TN = Tamil Nadu, TL = Telangana, TP = Tripura, UP = Uttar Pradesh, UK = Uttarakhand, WB = West Bengal, OD = Odisha, AP = Andhra Pradesh, JK = Jammu & Kashmir, LD = Ladakh.

Regional disparities in COVID-19 infection rates and adjusted death rates highlight variations in transmission intensity and mortality burden. Southern and western states experienced higher transmission rates than other regions, indicating geographical clustering of COVID-19 cases influenced by population density, mobility, and healthcare infrastructure. Age-adjusted death rates varied across states, with Goa reporting the highest death rate at 236 per 100,000, followed by Delhi (146), Maharashtra (111), and Uttarakhand (71). Uttar Pradesh had the lowest age-adjusted death rate at 13 per 100,000, reflecting variations in the pandemic’s severity across regions (Figure 2).

Similarly, DALY rates exhibited regional disparities. Goa had the highest DALY rate (3,071 per 100,000), followed by Delhi (2,263), Maharashtra (1,705), and Uttarakhand (1,056). The central region showed lower DALY rates, with Bihar recording the lowest DALY rate at 174 per 100,000, suggesting relatively lower disease burden in this region compared to southern and western states.

**Figure 3:**
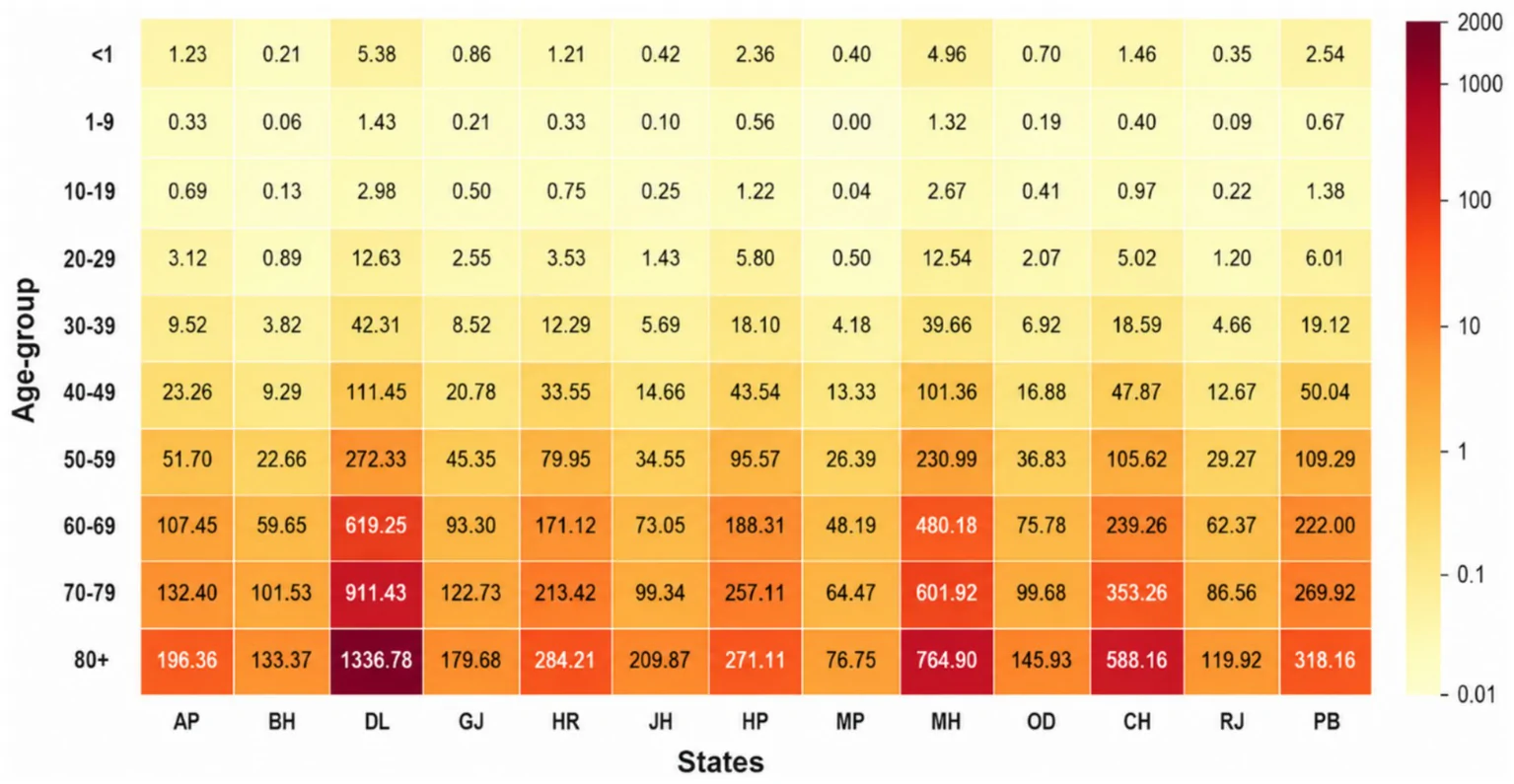
Age-specific COVID mortality rates across selected states. AP = Andhra Pradesh, BH = Bihar, DL = Delhi, GJ = Gujarat, HR = Haryana, JH = Jharkhand, HP = Himachal Pradesh, P = Madhya Pradesh, MH = Maharashtra, OD = Odisha, CH = Chhattisgarh, RJ = Rajasthan, PB = Punjab.

**Figure 4:**
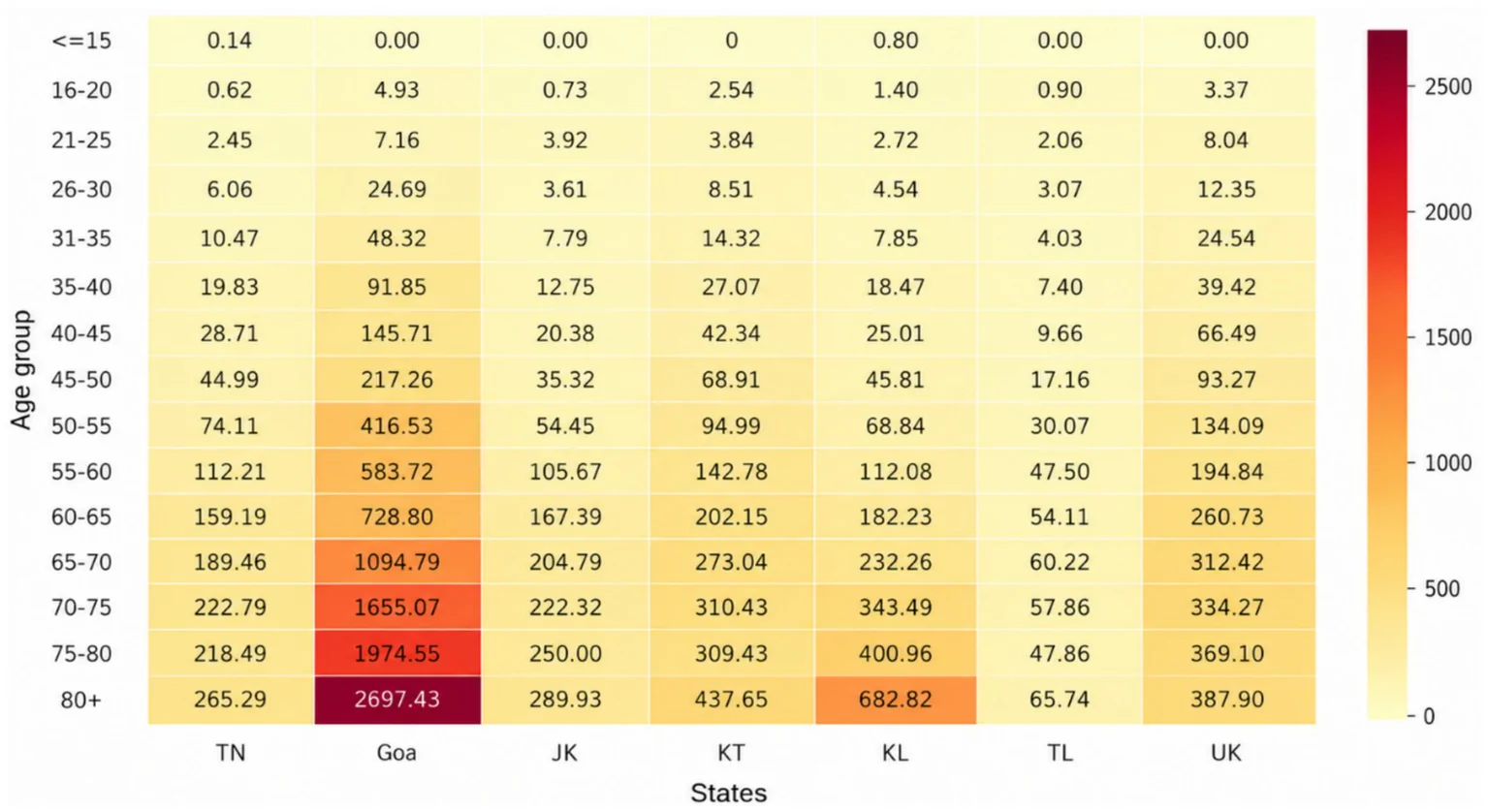
Age-specific COVID mortality rates across selected states. TN = Tamil Nadu, JK = Jammu and Kashmir, KT = Karnataka, KL = Kerala, TL = Telangana, UK = Uttarakhand.

Age-specific COVID-19 mortality distribution across states offers insights into the demographics of mortality. Maharashtra had the highest mortality across all age categories, peaking in the 60–69 age group with around 40,000 deaths. This pattern was broadly replicated across the other states, though the numbers were much lesser. Delhi saw about 5,000 deaths in this age group, with lower numbers in other states. Mortality data from seven states intervals allowed comparisons, revealing that Karnataka had the highest mortality (5,000 deaths) in the 65–70-year age group, while Uttar Pradesh had the younger mortality profile, peaking in the 60–65-year age group. Kerala’s mortality was concentrated among individuals aged over 60, highlighting the vulnerability of its older population (Figures 3, 4)

**Figure 5:**
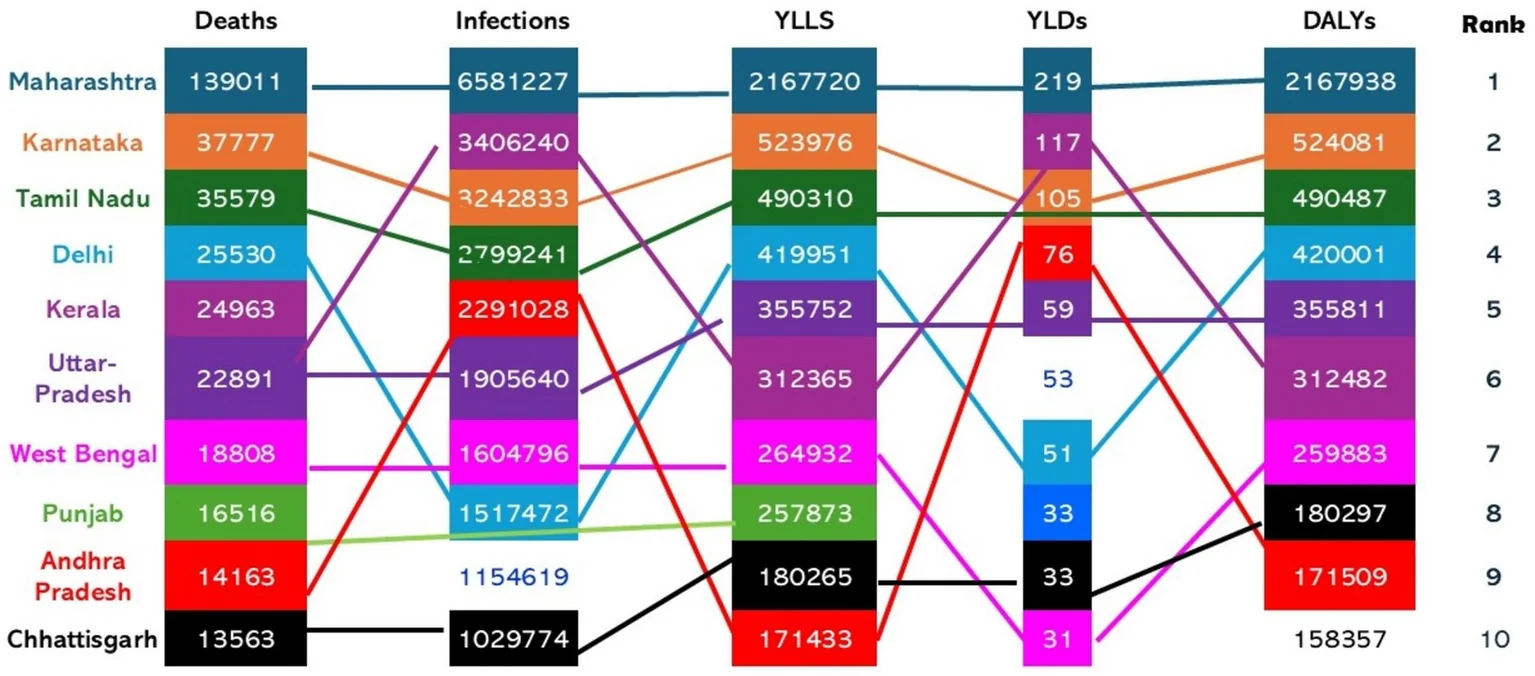
Top 10 states with COVID disease burden.

During the first and second waves of COVID-19, India lost 1.309 million years of potential productive life (YPPLL). Maharashtra recorded the highest (390,000 years) followed by Karnataka (110,000), Tamil Nadu (98,000) and Uttar Pradesh (90,000). Goa had the lowest YPPLL (9,147) years, indicating a relatively smaller impact.

India’s cumulative cost of productivity loss (CPL) due to COVID-19 amounted to ₹195.05 billion from premature mortality among the working-age population and ₹268.13 billion owing to absenteeism from work among infected individuals. Maharashtra incurred the highest economic burden due to premature mortality (₹588.9 billion) followed by Karnataka (₹202.7 billion), Tamil Nadu (₹173.7 billion), and Delhi (₹164 billion). Maharashtra also led in the absenteeism -related CPL (₹481.7 billion), followed by Karnataka (₹335.1 billion), Kerala (₹292.9 billion), and Tamil Nadu (₹269.5 billion). Jammu and Kashmir reported the lowest CPL from mortality (₹0.79 billion) while Bihar had the least absenteeism-related CPL (₹1.25 billion; Table 5).

**Table 5:** Comparison of YPPLL and cost of productivity loss among different states.

State	YPPLL (in person years)	CPL mortality (In ₹)	CPL due to absenteeism from work (In ₹)
All India	13,09,883	1,95,05,07,27,000	2,68,13,10,26,964
Andhra Pradesh	39,806	5,72,57,87,492	18,25,74,26,936
Assam	28,766	1,64,78,35,008	1,78,82,79,164
Bihar	27,150	81,17,97,202	1,25,50,25,841
Chhattisgarh	38,120	3,48,71,47,700	5,36,50,07,597
Delhi	71,754	16,40,33,22,191	18,48,29,86,048
Goa	9,147	2,83,45,27,201	2,70,85,74,539
Gujarat	28,370	4,61,95,43,484	8,81,97,41,855
Haryana	27,752	4,62,54,20,214	8,39,63,90,235
Himachal Pradesh	10,295	1,65,63,72,810	1,87,83,23,048
Jammu And Kashmir	11,446	79,23,87,004	1,32,75,94,444
Jharkhand	14,441	82,62,46,616	1,36,29,98,641
Karnataka	1,10,963	20,27,96,20,490	33,51,90,02,852
Kerala	42,518	6,07,35,02,935	29,29,24,45,360
Madhya Pradesh	35,685	3,01,67,04,978	4,64,64,74,074
Maharashtra	3,90,701	58,89,39,80,792	48,16,73,71,692
Odisha	23,041	1,79,89,29,159	4,01,41,74,433
Punjab	46,420	4,95,33,57,189	4,12,11,47,954
Rajasthan	25,166	2,39,23,18,060	5,12,24,25,994
Tamil Nadu	98,401	17,37,20,50,192	26,95,37,24,740
Telangana	17,904	3,40,48,15,294	1,40,19,77,813
Uttar-Pradesh	89,985	3,30,85,96,815	4,25,16,38,967
Uttarakhand	24,425	3,29,34,52,201	3,03,35,35,907
West Bengal	61,156	5,24,53,03,740	6,54,09,57,188
Other NE	27,899	3,57,77,53,460	2,80,64,04,905
Other UTs	8,572	5,94,12,45,011	9,80,56,93,340

Across all measures of disease and economic burden, Maharashtra ranked highest followed by southern states Karnataka, Kerala and Tamil Nadu and Andhra Pradesh. Among the northern states Delhi ranked the highest followed by Uttar Pradesh (Figure 5). Similar trend was observed for economic burden due to absenteeism from work and early mortality among the working population (Table 6).

**Table 6:** Top 10 states with higher cost of productivity loss.

Rank	State	CPL mortality (in million)	State	CPL absenteeism from work (in millions)
1	Maharashtra	58,894	Maharashtra	48,167
2	Karnataka	20,280	Karnataka	33,519
3	Tamil Nadu	17,372	Kerala	29,292
4	Delhi	16,403	Tamil Nadu	26,954
5	Kerala	6,074	Delhi	18,483
6	Other UTs	5,941	Andhra Pradesh	18,257
7	Andhra Pradesh	5,726	Other UTs	9,806
8	West Bengal	5,245	Gujarat	8,820
9	Punjab	4,953	Haryana	8,396
10	Haryana	4,625	West Bengal	6,541

The Supplementary material provides further detailed statistics on age and gender-wise impacts, on YLLs, YLDs, DALYs, YPPLL, and CPL providing a comprehensive demographic analysis of the pandemic’s impact.

## Discussion

The COVID-19 pandemic has revealed itself as one of the most significant global health crises, affecting various regions at multiple levels. Comparative studies from India, focusing on states like Kerala and Maharashtra, provide insights into the diverse impacts across different regions, as well as discrepancies in reported data. India’s healthcare system follows a federal structure, where responsibilities are shared between the central and state governments, often leading to variations in how services are delivered. This decentralization can result in differences in data collection practices, standards, and reporting practices (31–34) across states. As a result, compiling consistent nationwide data and coordinating a unified response can be challenging. Researchers and modelers utilize the available data to predict infection rates and inform policy decisions (35–41). Calculating premature mortality in terms of Years of Life Lost (YLL) is essential, particularly since COVID-19 affects individuals below the age of 60. Studies from different countries, including Korea (42), Italy (43), and India (44, 45), have provided insights into YLLs and DALYs associated with COVID-19. However, comprehensive DALY estimates for COVID-19 in India are still lacking, with most studies focusing on specific health states or using country-specific disability weights.

The Kerala study estimated around 3.41 million cases and 10,400 deaths by June 2021, while the current analysis shows 2.68 million cases and over 24,963 deaths, highlighting a significant difference likely due to different timeframes and data sources. These variations could be likely due to difference in the methodology, use of population projections for 2021 in our study versus their use of census data from 2011. Additionally, the disability weights in our analysis were based on broadly observable stages aligning with WHO severity definitions, based on a published paper unlike the granular stages employed in the Kerala paper based on an unpublished expert opinion. This difference in severity definitions, used for hospitalized patients in the COVID clinical registry, influenced the input data for disease duration and severity percentages, leading to higher estimates of economic and disease burden for Kerala in our study. Our findings indicate a more extensive effect on working-age populations, exacerbated by heightened premature mortality and absenteeism from work. The economic ramifications are evident in the Cost of Productivity Loss (CPL) due to mortality, amounting to ₹6,983,392 for males and ₹2,483,980 for females.

Our findings for Maharashtra align and diverge from the published study, presenting a comprehensive perspective on COVID-19’s impact across different age groups. Both studies report a substantial increase in Years of Life Lost (YLL) and a higher incidence of COVID-19 cases among younger age groups (20–49 years), with confirmed cases peaking in the 30–39 age group. However, our study documents a significantly higher total number of confirmed COVID-19 cases (6,581,277) and deaths (139,011) compared to Vasistha et al. (1,901,654 cases and 48,746 deaths). This disparity is likely due to the broader time frame and utilization of official data sources in our analysis.

In our study, the discounted YLL for the 50–59 age group stands at 539,159, indicating severe impacts on this demographic. In contrast, Vasistha et al. report an increased percentage share of Years of Potential Life Lost (YPLL) among working adults aged 45–65, rising from 33 to 50% due to COVID-19. The DALYs per 1,000 in our study was 17.05 compared to 11.57 reported by Vasistha et al.

When compared with findings from West Bengal (46) our study similarly demonstrated a higher burden of COVID-19 cases among the working-age population, particularly those aged 31–60 age group (1,604,796 cases and 18,808 deaths vs. 1,711,957 cases and 19,864 deaths). The comparable age-wise distribution indicates similar transmission patterns across age cohorts in West Bengal. Both studies also identified substantial productivity losses among older working -age adults especially in the 46–60-year age group. However, differences in the estimated economic burden were observed reflecting methodological variations between studies. Both studies, nonetheless, consistently demonstrate the greater productivity losses among males across all age groups, highlighting the gender disparity in the impact due to COVID-19’s. The COVID-19 pandemic has disproportionately affected older individuals, with adults over 65 years representing 80% of hospitalizations and having a 23-fold greater risk of death compared to those under 65 (47). This indicates that older adults, especially those above 40, may experience more severe illness and prolonged disability due to physical deterioration and comorbidities such as cardiovascular disease, diabetes, and obesity. The extended lockdowns and restrictions on movements limited in-person counselling and exacerbated health issues among the older adults above 60 years (48).

Although our study did not directly assess the risk of hospitalization, studies have shown that unvaccinated adults had a higher risk of moderate to severe, critical, or fatal COVID-19 (OR 1.54; 95% CI 1.09–2.16) and an increased risk of COVID-19-associated mortality (OR 1.80; 95% CI 1.10–2.87) compared to vaccinated individuals (49).

The observed gender disparity in COVID-19 burden may be explained by a combination of biological and behavioural factors. Biologically, males have been shown to exhibit differential immune responses, including lower innate and adaptive immune activation compared to females, which may increase susceptibility to severe outcomes. Additionally, a higher prevalence of comorbidities such as cardiovascular disease, hypertension, and diabetes among males may further elevate mortality risk.

From a behavioural perspective, occupational exposure, higher mobility patterns, and greater participation in the workforce during the pandemic likely increased infection risk among males. Differences in health-seeking behaviour and delayed access to care may also contribute to worse outcomes. These findings highlight the need for gender-responsive public health strategies that account for both biological vulnerability and social determinants of exposure.

The substantial variation in age-adjusted death rates across states such as the markedly higher rates observed in Goa compared to Uttar Pradesh can be attributed to multiple interrelated factors. Differences in health system capacity, including availability of hospital beds, intensive care units, and oxygen support, likely influenced survival outcomes during peak transmission periods. States with more robust health infrastructure and better reporting systems may also exhibit higher recorded mortality due to more complete detection. Testing strategies and surveillance intensity further contribute to these differences. States with widespread testing, including asymptomatic and high-risk populations, are more likely to detect infections and attribute deaths accurately to COVID-19. In contrast, limited testing capacity may result in under-ascertainment of both cases and deaths. Demographic structure also plays a critical role. States such as Goa, with a relatively older population, are more vulnerable to higher mortality, whereas states like Uttar Pradesh have a younger age distribution, potentially reducing overall mortality rates. Additionally, the prevalence of comorbid conditions, urbanization levels, population density, and mobility patterns may collectively shape transmission dynamics and disease severity across states.

During 2021, India’s GDP was approx. ₹236.5 trillion. Though the economic losses due to premature mortality and absenteeism from work was 0.083 and 0.114% respectively, these represent lost productive years or long-term economic capacity concentrated in working-age populations, amplifying their impact beyond the raw percentage affecting many sectors simultaneously. It must be also stated that these figures do not reflect the healthcare costs, long COVID productivity losses, job losses and business disruptions. To contextualize these estimates, the combined productivity losses represent a substantial economic burden when considered relative to national and state economies. Although these losses constitute a modest proportion of India’s gross domestic product (GDP), they reflect significant disruptions to the working-age population and labour productivity. India’s health expenditure during 2021 was ₹9,04,461 crore and the combined impact of the productivity losses due to premature mortality and absenteeism from work accounted for about 5–6% of India’s total health expenditure signalling a significant indirect cost of the pandemic, with implications for prioritizing investments in public health resilience and workforce protection. The losses from premature deaths and absenteeism during COVID-19 go beyond one-time economic shocks, affecting people in their most productive years. This means fewer workers, lost skills, and disruptions that can slow growth over time. Even if small as a share of GDP, these impacts can have lasting effects on the economy’s future potential.

This study, while comprehensive, acknowledges limitations. A significant constraint pertains to the assumptions made to infer death distributions for certain states lacking detailed, age-specific mortality data. Despite rigorous efforts to obtain reliable data from governmental sources, data gaps necessitated reliance on estimations that may influence the overall precision of the disease burden assessment.

A critical consideration in interpreting these findings is the potential under-ascertainment of COVID-19 cases and deaths in India. Despite the use of nationally aggregated datasets, the reported burden of approximately 34 million confirmed cases likely underestimates the true scale of infection, as suggested by seroprevalence studies and excess mortality analyses. Underreporting may arise from limited testing capacity during early pandemic phases, differential access to healthcare, and variability in state-level surveillance systems.

Importantly, such under-ascertainment has direct implications for burden of disease estimates. While mortality-based metrics such as Years of Life Lost (YLL) may be relatively less sensitive to underreporting compared to incidence-based measures, Years Lived with Disability (YLD) and overall DALYs may be underestimated due to incomplete capture of mild and asymptomatic infections. Consequently, the estimates presented in this study should be interpreted as conservative approximations of the true health and economic burden.

However, the use of standardized national data sources ensures internal consistency and comparability across states, which remains essential for policy prioritisation. Future studies incorporating excess mortality estimates and serological data could further refine these burden estimates and provide a more comprehensive assessment of pandemic impact. Importantly, only the COVID-19 test reports issued by these registered laboratories were recognized as legitimate and valid for any medical or administrative claims, including diagnostic confirmation and reporting purposes. This ensured the authenticity and reliability of the testing data used for the study. By leveraging this comprehensive national database, the analysis minimized discrepancies and inaccuracies that could arise from unregistered or unverified testing sources, thereby maintaining the credibility and accuracy of the findings.

The disparity in the number of reported COVID-19 cases between states can be attributed to several demographic, health system, epidemiological, and policy-related factors. Some states consistently conducted a higher number of tests per capita employing systematic and widespread testing that included symptomatic, asymptomatic, and high-risk populations while others concentrated primarily on symptomatic individuals or close contacts. So these states’ health surveillance system, captured cases more comprehensively.

Population density and urbanization also played a significant role. However, the present analysis does not explicitly differentiate between rural and urban populations due to the lack of consistently available disaggregated data across states. Given the substantial differences in healthcare access, population density, occupational structure, and exposure risk between rural and urban areas in India, the pandemic experience is likely to vary significantly across these settings. Future studies incorporating finer spatial resolution data would provide more nuanced insights into these disparities and support more targeted public health interventions. States with predominantly rural population, experienced relatively lower rates of virus transmission due to lower population density in rural areas. Whereas states with a higher degree of urbanization and densely packed cities, witnessed faster spread of the virus, particularly in crowded urban areas. Well-established healthcare infrastructure and proactive disease surveillance enabled early detection and reporting of COVID-19 cases in some states whereas in those states with limited healthcare access and infrastructure, especially in rural areas, led to many cases going undetected. Differences in health-seeking behaviour further contributed to this difference.

Policy implementation and pandemic response also influenced case numbers. States like Kerala implemented strong public health measures early in the pandemic, including rigorous contact tracing, isolation, and quarantine protocols, ensuring higher detection rates.

Migration and mobility patterns were another critical factor. Demographics and socioeconomic factors also played a role. States with a higher proportion of older adults above 60 years, who are more vulnerable to symptomatic infection and complications, leading to higher testing and case detection rates. Additionally, the dynamics of the disease and the emergence of variants influenced the positivity rates. Vaccination campaigns also played a role.

The findings of this study have important implications for targeted policy interventions and resource allocation. High-burden states such as Maharashtra, Karnataka, and Tamil Nadu require strengthened health system capacity, including expansion of critical care infrastructure, workforce augmentation, and improved disease surveillance systems. Investments in early detection, testing, and rapid response mechanisms are essential to reduce transmission and mortality during future outbreaks. In contrast, states with lower reported burden but potential under-ascertainment, such as Uttar Pradesh and Bihar, may benefit from strengthening surveillance systems, improving reporting accuracy, and expanding access to diagnostic services. Tailored strategies that account for demographic structure, comorbidity prevalence, and healthcare accessibility are critical for effective pandemic response. While this study focuses on quantifying health burden and productivity losses associated with COVID-19, it does not explicitly capture other important dimensions of socioeconomic impact, including healthcare expenditure (both out-of-pocket and public), disruptions to education, and caregiver burden. These components represent significant indirect costs of the pandemic and may further amplify its overall societal impact. However, the inclusion of these dimensions requires detailed and longitudinal data that are not consistently available across states. Therefore, the present analysis should be interpreted as a partial but policy-relevant assessment of the broader socioeconomic burden. From an economic perspective, prioritizing interventions that reduce premature mortality among the working-age population can yield substantial gains in productivity and economic stability. Allocating resources based on state-specific burden estimates, as demonstrated in this study, can enhance the efficiency and equity of public health investments. These findings underscore the need for a data-driven, decentralized approach to pandemic preparedness and health system strengthening in India.

## Conclusion

This study provides a comprehensive and policy-relevant assessment of the health and economic burden of COVID-19 across Indian states using nationally representative datasets and a standardized burden of disease framework. By integrating estimates of Years of Life Lost (YLL), Years Lived with Disability (YLD), Disability-Adjusted Life Years (DALYs), and Years of Potential Productive Life Lost (YPPLL), the analysis offers a robust and comparable evaluation of pandemic impacts across diverse demographic and epidemiological contexts. The findings reveal substantial heterogeneity in both health and economic burden across states, reflecting differences in population structure, healthcare capacity, surveillance systems, and pandemic response strategies. High-burden states such as Maharashtra, Karnataka, and Kerala demonstrate the need for targeted strengthening of health system resilience, while lower-burden states highlight potential gaps in detection, reporting, or differential exposure patterns. These insights underscore the importance of state-specific, data-driven policy interventions rather than uniform national approaches. Importantly, the methodological framework developed in this study emphasizes transparency in data integration, explicit acknowledgment of assumptions, and careful interpretation of uncertainty. While the estimates presented are subject to limitations related to data availability and reporting variability, they provide a consistent and policy-relevant basis for prioritizing interventions and resource allocation. From a policy perspective, the results highlight the need for strengthening integrated disease surveillance systems, improving real-time access to disaggregated health data, and incorporating economic burden metrics into pandemic preparedness planning. Investments in health infrastructure, targeted protection of high-risk populations, and adaptive response strategies tailored to state-specific contexts are critical for mitigating the impact of future public health emergencies. Overall, this study contributes to the growing evidence base on pandemic burden estimation and provides a scalable analytical framework that can be applied in other low- and middle-income settings with similar data constraints. Strengthening data systems and embedding burden of disease approaches into routine health planning will be essential for enhancing preparedness, response, and resilience in future pandemics.

## Limitation

First, the analysis is restricted to the period from March 2020 to September 2021, encompassing the first and second waves of the COVID-19 pandemic in India. Subsequent waves, including those driven by emerging variants such as Omicron, are not captured in this study. Given the evolving epidemiological characteristics, vaccination coverage, and healthcare responses in later phases, the estimates presented here may not fully reflect the cumulative burden of COVID-19 in India. Second, the analysis provides COVID-19 burden estimates for most large states where complete data were available. However, for the northeastern states (excluding Assam), population projections from the Registrar General of India (RGI) were available only in aggregated form, and age-specific life expectancy estimates were not accessible. Consequently, estimates for seven northeastern states were derived in a combined manner. These states collectively accounted for approximately 1.3% of the total reported COVID-19 cases and deaths in India, thereby limiting their influence on national-level estimates. Similarly, for union territories, population projections and age- and sex-specific data were available only in aggregated formats. The combined reported cases and deaths from Andaman and Nicobar Islands, Dadra and Nagar Haveli, Daman and Diu, Ladakh, Lakshadweep, Puducherry, and Chandigarh accounted for less than 1% of total cases and deaths. Where disaggregated data were available, such as for Goa and Jammu and Kashmir, separate estimates were provided.

Third, the study does not account for post-COVID-19 conditions (long COVID) or long-term functional impairments due to the lack of reliable longitudinal data. As a result, the Years Lived with Disability (YLD) and overall DALY estimates are likely to underestimate the true burden of disease. Fourth, the analysis does not explicitly account for multiple infections per individual over the study period. Reinfections, particularly during later phases of the pandemic, may contribute to additional absenteeism from work and productivity losses that are not captured in the current estimates. Fifth, potential inaccuracies in mortality reporting, including underreporting and possible misclassification of COVID-19 deaths, may influence the precision of Years of Life Lost (YLL) estimates. Although efforts were made to align reported deaths with official counts, residual bias, including both undercounting and possible double counting in certain contexts, cannot be entirely excluded. Sixth, the severity distribution and hospitalization patterns were derived from the National Clinical Registry for COVID-19 (NCRC), which may be subject to selection bias, as it primarily captures cases from participating hospitals and may not fully represent all healthcare settings across states. Seventh, the analysis does not explicitly adjust for variations in vaccination coverage during the study period. The phased rollout of COVID-19 vaccines in India may have influenced disease severity, hospitalization, and mortality patterns. Eighth, temporal variations in circulating SARS-CoV-2 variants, including Alpha and Delta, are not explicitly incorporated into the analysis. Differences in transmissibility and virulence associated with these variants may affect both morbidity and mortality estimates. Finally, the study focuses primarily on health burden and productivity losses and does not incorporate other indirect socioeconomic impacts such as healthcare expenditure, education disruption, and caregiver burden. Additionally, the analysis does not differentiate between rural and urban populations due to the lack of consistent disaggregated data. These factors may further influence the overall societal burden and should be considered when interpreting the findings.

## Data Availability

The data supporting the findings of this study are available within the article and its supplementary materials. Further inquiries can be directed to the corresponding authors.
