# Correction: A state wise analysis of the socioeconomic and health impacts of the COVID-19 pandemic in India: lessons for future health system preparedness

**DOI:** 10.3389/fpubh.2026.1954305

**Published:** 2026-08-20

**Authors:** Geetha R. Menon, U. Venkatesh, Jeetendra Yadav, Krushna Chandra Sahoo, Tanu Anand, Aparna Mukherjee, Gunjan Kumar, Alka Turuk, Ashoo Grover, Saurabh Sharma, Sandhya Singh, Firoz Khan

**Affiliations:** 1 Indian Council of Medical Research, New Delhi, India; 2 Department of Community Medicine & Family Medicine, All India Institute of Medical Sciences, Gorakhpur, Uttar Pradesh, India; 3 ICMR-National Institute for Research in Digital Health and Data Science, (Formerly, ICMR-National Institute of Medical Statistics), Medical Enclave Ansari Nagar, New Delhi, India; 4 Health Technology Assessment in India (HTAIn), Department of Health Research, Ministry of Health & Family Welfare, Govt. of India, New Delhi, India; 5 Clinical Studies & Trials Unit (CSTU), Division of Development Research, ICMR, New Delhi, India; 6 Division of Delivery, ICMR, New Delhi, India; 7 Social Statistics Division, National Statistical Office, Ministry of Statistics and PI, Govt of India, New Delhi, India

**Keywords:** cost of productivity life, COVID-19, disability-adjusted life years, GDP, years of potential productivity life lost

“The pandemic has…” needs to be replaced by “The pandemic had…”.

A correction has been made to the section Abstract, paragraph 4, section-Interpretation.. It now reads: “The pandemic had serious economic consequences, particularly for the working-age population, resulting in lost productivity from illness or death.”

The following repeated text needs to be removed from the middle of the paragraph: “As a result, there remains a critical gap in nationally representative analyses that simultaneously quantify mortality, morbidity, and productivity losses using standardized metrics such as DALYs and Years of Potential Productive Life Lost (YPPLL).” A correction has been made to the section Introduction, paragraph 4. It now reads in full as follows:

“The COVID-19 pandemic was one of the most critical challenges, with significant consequences for individuals, society, and the national economy. While individuals suffered from health issues and loss of productivity, the national economy experienced reduced output and higher unemployment rates. Numerous studies have explored the health and socioeconomic impact of COVID-19 in India, though most rely on small or localized datasets. These studies, however, have predominantly focused on localized populations, specific clinical outcomes, or short-term impacts, and often lack integration of both health and economic burden within a unified analytical framework. Moreover, few studies have applied standardized burden of disease methodologies to generate comparable state-level estimates across India. However, no studies have provided a comprehensive socioeconomic analysis of COVID-19's health effects using nationally representative datasets. As a result, there remains a critical gap in nationally representative analyses that simultaneously quantify mortality, morbidity, and productivity losses using standardized metrics such as DALYs and Years of Potential Productive Life Lost (YPPLL).”

The original version of this article has been updated.

