# Supplementary material for: A state wise analysis of the socioeconomic and health impacts of the COVID-19 pandemic in India: lessons for future health system preparedness
Source: Front Public Health. 2026 Jul 21;14:1710029. doi: 10.3389/fpubh.2026.1710029 (PMC13435320; doi:10.3389/fpubh.2026.1710029)
Supplement: Supplementary file 1 [file Supplementary_file_1.DOCX]

| Table S1: Age-specific Health and Economic Burden Indicators Associated with COVID-19-Andhra Pradesh | | | | | | | | | | | | | | |  | |  | |  | |
| --- | --- | --- | --- | --- | --- | --- | --- | --- | --- | --- | --- | --- | --- | --- | --- | --- | --- | --- | --- | --- |
| Age group | **Deaths** | | | **YLL discounted** | | | | **YLD** | | **DALYS** | | | **CPL due to premature mortality** | **CPL due to absenteeism from work** | | **CPL due to premature mortality** | | **CPL due to absenteeism from work** | |  |
|  | **Male** | **Female** | **Both** | **Male** | **Female** | **Both** | **Male** | **Female** | **Both** | **Male** | **Female** | **Both** | **Male** |  | | **Female** | |  | |  |
| <1 | 5 | 4 | 9 | 140 | 112 | 252 | 0.003 | 0.002 | 0.005 | 139.53 | 112.18 | 251.71 |  |  | |  | |  | |  |
| 1-9 | 13 | 8 | 21 | 393 | 224 | 617 | 1.033 | 0.990 | 2.023 | 393.96 | 224.57 | 618.53 |  |  | |  | |  | |  |
| 10-19 | 29 | 25 | 54 | 773 | 670 | 1443 | 3.356 | 3.220 | 6.576 | 776.06 | 673.15 | 1449.21 | 2,519,797 | 113,265,955 | | 1,256,478 | | 60,935,972 | |  |
| 20-29 | 176 | 108 | 284 | 4223 | 2674 | 6897 | 6.947 | 6.034 | 12.981 | 4230.32 | 2679.79 | 6910.11 | 181,778,802 | 2,862,486,447 | | 56,600,128 | | 1,059,886,932 | |  |
| 30-39 | 608 | 239 | 847 | 12856 | 5236 | 18091 | 8.238 | 6.449 | 14.687 | 12864.07 | 5242.10 | 18106.17 | 944,141,360 | 4,048,690,927 | | 239,531,870 | | 1,709,874,910 | |  |
| 40-49 | 1189 | 555 | 1744 | 21021 | 10277 | 31298 | 8.597 | 7.275 | 15.873 | 21029.55 | 10284.72 | 31314.27 | 1,744,169,071 | 3,266,278,227 | | 525,924,519 | | 1,655,496,039 | |  |
| 50-59 | 1942 | 1033 | 2975 | 27009 | 14957 | 41966 | 7.047 | 5.829 | 12.876 | 27016.22 | 14962.56 | 41978.78 | 1,568,083,965 | 2,425,211,118 | | 461,781,501 | | 1,055,300,409 | |  |
| 60-69 | 2501 | 1402 | 3903 | 25027 | 14708 | 39735 | 4.230 | 3.386 | 7.617 | 25031.53 | 14710.91 | 39742.45 |  |  | |  | |  | |  |
| 70-79 | 1952 | 981 | 2933 | 12794 | 7361 | 20155 | 1.581 | 1.226 | 2.807 | 12795.57 | 7362.29 | 20157.86 |  |  | |  | |  | |  |
| 80+ | 934 | 460 | 1394 | 6934 | 4045 | 10979 | 0.292 | 0.244 | 0.536 | 6934.44 | 4045.13 | 10979.57 |  |  | |  | |  | |  |
| Total | **9349** | **4814** | **14163** | **111170** | **60263** | **171433** | **41** | **35** | **76** | **111211** | **60297** | **171509** | **4,440,692,994** | **12,715,932,673** | | **1,285,094,497** | | **5,541,494,262** | |  |

| Table S2: Age-specific Health and Economic Burden Indicators Associated with COVID-19-Assam | | | | | | | | | | | | | | | | | | | | | | |  |  | |  | |  | |  |
| --- | --- | --- | --- | --- | --- | --- | --- | --- | --- | --- | --- | --- | --- | --- | --- | --- | --- | --- | --- | --- | --- | --- | --- | --- | --- | --- | --- | --- | --- | --- |
| Age group | **Deaths** | | | **YLL discounted** | | | | | | | | **YLD** | | | | | **DALYS** | | | | | | **CPL due to premature mortality** | | **CPL due to absenteeism from work** | | **CPL due to premature mortality** | | **CPL due to absenteeism from work** | |
|  | **Male** | **Female** | **Both** | **Male** | | **Female** | | **Both** | | **Male** | | | **Female** | | **Both** | | | **Male** | | **Female** | | **Both** | **Male** | | | **Female** | | | |  |
| <1 | 8 | 4 | 12 | 238 | 102 | | 340 | | 0.035 | | 0.032 | | | 0.067 | | 237.71 | | | 102.49 | | 340.21 | |  | |  | |  | |  |  |
| 1-15' | 8 | 4 | 12 | 239 | 103 | | 343 | | 0.624 | | 0.60 | | | 1.221 | | 239.90 | | | 103.89 | | 343.79 | |  | |  | |  | |  |  |
| 16-20 | 25 | 11 | 35 | 666 | 290 | | 955 | | 0.495 | | 0.47 | | | 0.963 | | 666.22 | | | 290.06 | | 956.29 | | 3587388.394 | | 27797989.9 | | 416692.6439 | | 7014595.056 |  |
| 21-30 | 288 | 123 | 411 | 7490 | 3268 | | 10758 | | 2.025 | | 1.73 | | | 3.755 | | 7492.38 | | | 3269.43 | | 10761.81 | | 155285862.1 | | 424749785.2 | | 16025903.29 | | 79394177.09 |  |
| 31-45 | 987 | 423 | 1410 | 23362 | 10273 | | 33635 | | 3.462 | | 2.51 | | | 5.972 | | 23365.24 | | | 10275.48 | | 33640.72 | | 661822868.2 | | 658353146.2 | | 63222332.18 | | 109069353.6 |  |
| 46-60 | 1274 | 546 | 1821 | 24251 | 10804 | | 35056 | | 2.934 | | 2.06 | | | 4.991 | | 24254.36 | | | 10806.23 | | 35060.58 | | 827138889.4 | | 403186618.3 | | 84780189.53 | | 78713498.29 |  |
| 61-75 | 1028 | 440 | 1468 | 13625 | 6126 | | 19751 | | 1.163 | | 0.77 | | | 1.934 | | 13626.04 | | | 6127.07 | | 19753.11 | |  | |  | |  | |  |  |
| 75+ | 493 | 211 | 705 | 3778 | 1716 | | 5493 | | 0.256 | | 0.17 | | | 0.422 | | 3777.85 | | | 1715.98 | | 5493.82 | |  | |  | |  | |  |  |
| Total | **4111** | **1762** | **5873** | **73649** | **32682** | | **106331** | | **11** | | **8** | | | **19** | | **73660** | | | **32691** | | **106350** | | **1,647,835,008** | | **1,514,087,540** | | **164,445,118** | | **274,191,624** |  |

| Table S3: Age-specific Health and Economic Burden Indicators Associated with COVID-19-Bihar | | | | | | | | | | | | | | |  | |  | | |  | |
| --- | --- | --- | --- | --- | --- | --- | --- | --- | --- | --- | --- | --- | --- | --- | --- | --- | --- | --- | --- | --- | --- |
| Age group | **Deaths** | | | **YLL discounted** | | | | **YLD** | | **DALYS** | | | **CPL due to premature mortality** | | **CPL due to absenteeism from work** | | **CPL due to premature mortality** | | | **CPL due to absenteeism from work** | |
|  | **Male** | **Female** | **Both** | **Male** | **Female** | **Both** | **Male** | **Female** | **Both** | **Male** | **Female** | **Both** | **Male** |  | | **Female** | |  | | |  |
| <1 | 3 | 3 | 6 | 95 | 75 | 171 | 0.005 | 0.002 | 0.007 | 95.30 | 75.42 | 170.71 |  |  | |  | |  | | |  |
| 1-9 | 9 | 5 | 14 | 269 | 151 | 420 | 0.306 | 0.261 | 0.568 | 269.08 | 151.26 | 420.34 |  |  | |  | |  | | |  |
| 10-19 | 20 | 17 | 37 | 565 | 479 | 1044 | 1.061 | 0.765 | 1.826 | 566.12 | 479.83 | 1045.95 | 349,876 | 9,937,531 | | 72,157 | | 1,199,300 | | |  |
| 20-29 | 120 | 74 | 194 | 3178 | 1948 | 5126 | 2.507 | 1.636 | 4.143 | 3180.03 | 1949.80 | 5129.84 | 24,932,022 | 266,939,959 | | 1,539,187 | | 11,256,398 | | |  |
| 30-39 | 415 | 163 | 578 | 10058 | 3931 | 13989 | 2.732 | 1.538 | 4.270 | 10060.80 | 3932.55 | 13993.35 | 159,438,209 | 393,717,441 | | 7,983,510 | | 19,548,919 | | |  |
| 40-49 | 811 | 379 | 1190 | 17282 | 8019 | 25301 | 2.698 | 1.579 | 4.277 | 17284.87 | 8020.43 | 25305.30 | 296,829,893 | 298,080,681 | | 27,731,530 | | 27,017,032 | | |  |
| 50-59 | 1325 | 704 | 2029 | 23295 | 12153 | 35449 | 2.160 | 1.310 | 3.469 | 23297.41 | 12154.66 | 35452.06 | 273,883,326 | 213,695,182 | | 19,037,492 | | 13,633,398 | | |  |
| 60-69 | 1706 | 956 | 2662 | 22509 | 12326 | 34835 | 1.391 | 0.818 | 2.209 | 22510.62 | 12326.51 | 34837.13 |  |  | |  | |  | | |  |
| 70-79 | 1331 | 669 | 2000 | 11302 | 5431 | 16732 | 0.469 | 0.230 | 0.699 | 11302.08 | 5430.82 | 16732.90 |  |  | |  | |  | | |  |
| 80+ | 637 | 314 | 951 | 2626 | 1211 | 3837 | 0.148 | 0.080 | 0.228 | 2625.65 | 1211.32 | 3836.97 |  |  | |  | |  | | |  |
| Total | **6376** | **3284** | **9660** | **91178** | **45724** | **136903** | **13** | **8** | **22** | **91192** | **45733** | **136925** | **755,433,326** | **1,182,370,794** | | **56,363,876** | | | **72,655,048** | |  |

| Table S4 Age-specific Health and Economic Burden Indicators Associated with COVID-19-Chattisgarh | | | | | | | | | | | | |  | |  | |  |  | |  | |  |
| --- | --- | --- | --- | --- | --- | --- | --- | --- | --- | --- | --- | --- | --- | --- | --- | --- | --- | --- | --- | --- | --- | --- |
| **Age group** | **Deaths** | | | **YLL discounted** | | | | **YLD discounted** | | | **DALYS** | | | | | **CPL due to premature mortality** | | | **CPL due to absenteeism from work** | **CPL due to premature mortality** | **CPL due to absenteeism from work** | |
|  | **Male** | **Female** | **Both** | **Male** | **Female** | **Both** | **Male** | | **Female** | **Both** | **Male** | **Female** | | **Both** | | **Male** | | | | **Female** | | |
| **<1** | 5 | 4 | 8 | 130 | 105 | 235 | 0.023 | | 0.02 | 0.04 | 130.12 | 105.00 | | 235.12 | |  | | |  |  |  | |
| **1-9** | 13 | 7 | 20 | 368 | 210 | 579 | 0.599 | | 0.56 | 1.16 | 368.95 | 210.96 | | 579.91 | |  | | | | | | |
| **10-19** | 28 | 24 | 52 | 768 | 666 | 1434 | 1.529 | | 1.44 | 2.97 | 769.53 | 667.23 | | 1436.76 | | 2,137,996 | | | 47,100,959 | 1,559,525 | 34,655,921 | |
| **20-29** | 168 | 103 | 272 | 4267 | 2698 | 6965 | 3.477 | | 3.04 | 6.52 | 4270.44 | 2700.98 | | 6971.42 | | 111,459,501 | | | 909,553,555 | 42,446,024 | 411,230,955 | |
| **30-39** | 583 | 228 | 811 | 13320 | 5436 | 18756 | 3.974 | | 3.01 | 6.99 | 13323.66 | 5438.92 | | 18762.59 | | 540,098,890 | | | 1,191,496,663 | 157,346,052 | 537,231,122 | |
| **40-49** | 1138 | 532 | 1670 | 22474 | 11062 | 33535 | 3.657 | | 2.73 | 6.38 | 22477.42 | 11064.44 | | 33541.86 | | 1,005,360,780 | | | 890,019,183 | 367,459,535 | 439,948,814 | |
| **50-59** | 1860 | 989 | 2849 | 29660 | 16823 | 46484 | 2.837 | | 2.06 | 4.90 | 29663.02 | 16825.47 | | 46488.49 | | 922,615,154 | | | 633,707,506 | 336,664,244 | 270,062,919 | |
| **60-69** | 2395 | 1342 | 3737 | 27848 | 17470 | 45318 | 1.467 | | 1.09 | 2.55 | 27849.24 | 17470.96 | | 45320.20 | |  | | |  |  |  | |
| **70-79** | 1869 | 940 | 2808 | 13439 | 7907 | 21346 | 0.481 | | 0.33 | 0.82 | 13439.19 | 7907.70 | | 21346.89 | |  | | | | | | |
| **80+** | 894 | 441 | 1335 | 3529 | 2085 | 5614 | 0.173 | | 0.12 | 0.29 | 3529.26 | 2084.76 | | 5614.02 | |  | | | | | | |
| **Total** | **8953** | **4610** | **13563** | **115803** | **64462** | **180265** | **18.218** | | **14.40** | **32.62** | **115820.85** | **64476.42** | | **180297.27** | | **2,581,672,319** | | | **3,671,877,866** | **905,475,380** | **1,693,129,731** | |

| Table S5: Age-specific Health and Economic Burden Indicators Associated with COVID-19-Delhi | | | | | | | | | | |  |  |  |  |  |  |
| --- | --- | --- | --- | --- | --- | --- | --- | --- | --- | --- | --- | --- | --- | --- | --- | --- |
| **Age group** | **Deaths** | | | **YLL discounted** | | | | **YLD** | | **DALYS** | | | **CPL due to premature mortality** | **CPL due to absenteeism from work** | **CPL due to premature mortality** | **CPL due to absenteeism from work** |
|  | **Male** | **Female** | **Both** | **Male** | **Female** | **Both** | **Male** | **Female** | **Both** | **Male** | **Female** | **Both** | **Male** |  | **Female** |  |
| <1 | 9 | 7 | 15 | 257 | 206 | 463 | 0.057 | 0.044 | 0.101 | 256.61 | 206.23 | 462.85 |  |  |  |  |
| 1-9 | 24 | 14 | 38 | 720 | 410 | 1130 | 0.536 | 0.488 | 1.023 | 720.21 | 410.32 | 1130.54 |  |  |  |  |
| 10-19 | 53 | 45 | 98 | 1518 | 1311 | 2829 | 1.501 | 1.326 | 2.827 | 1519.72 | 1311.89 | 2831.61 | 14,527,039 | 180,756,127 | 318,372 | 3,470,225 |
| 20-29 | 317 | 195 | 512 | 8584 | 5407 | 13991 | 4.489 | 3.847 | 8.336 | 8588.79 | 5410.43 | 13999.22 | 566,228,103 | 3,246,364,487 | 87,653,427 | 570,383,836 |
| 30-39 | 1097 | 430 | 1527 | 27440 | 11137 | 38577 | 5.718 | 4.367 | 10.086 | 27446.05 | 11140.91 | 38586.96 | 3,302,298,758 | 5,540,894,574 | 269,691,320 | 709,533,451 |
| 40-49 | 2143 | 1001 | 3144 | 47821 | 23410 | 71231 | 5.438 | 4.196 | 9.634 | 47826.00 | 23414.49 | 71240.49 | 6,155,179,591 | 4,237,003,799 | 431,672,393 | 422,749,106 |
| 50-59 | 3500 | 1862 | 5362 | 66159 | 37570 | 103729 | 5.062 | 4.131 | 9.193 | 66163.61 | 37574.33 | 103737.93 | 5,086,271,804 | 3,153,415,586 | 489,481,384 | 418,414,859 |
| 60-69 | 4508 | 2527 | 7035 | 67302 | 41470 | 108772 | 3.274 | 2.647 | 5.921 | 67305.01 | 41472.55 | 108777.56 |  |  |  |  |
| 70-79 | 3518 | 1769 | 5286 | 37629 | 21811 | 59440 | 1.450 | 1.151 | 2.601 | 37630.82 | 21811.77 | 59442.59 |  |  |  |  |
| 80+ | 1683 | 830 | 2513 | 12499 | 7291 | 19791 | 0.472 | 0.361 | 0.833 | 12499.86 | 7291.61 | 19791.46 |  |  |  |  |
| **Total** | **16852** | **8678** | **25530** | **269929** | **150022** | **419951** | **28** | **23** | **51** | **269957** | **150045** | **420001** | **15,124,505,295** | **16,358,434,572** | **1,278,816,896** | **2,124,551,476** |

| Table S6: Age-specific Health and Economic Burden Indicators Associated with COVID-19-Goa | | | | | | | | | | |  |  |  |  |  |  |
| --- | --- | --- | --- | --- | --- | --- | --- | --- | --- | --- | --- | --- | --- | --- | --- | --- |
| **Age group** | **Deaths** | | | **YLL discounted** | | | | **YLD** | | **DALYS** | | | **CPL due to premature mortality** | **CPL due to absenteeism from work** | **CPL due to premature mortality** | **CPL due to absenteeism from work** |
|  | **Male** | **Female** | **Both** | **Male** | **Female** | **Both** | **Male** | **Female** | **Both** | **Male** | **Female** | **Both** | **Male** |  | **Female** |  |
| **<1** | 0 | 0 | 0 | 0 | 0 | 0 | 0.009 | 0.007 | 0.017 | 0.01 | 0.01 | 0.02 |  |  |  |  |
| **1-5** | 0 | 0 | 0 | 0 | 0 | 0 | 0.061 | 0.057 | 0.118 | 0.06 | 0.06 | 0.12 |  |  |  |  |
| **6-10** | 0 | 0 | 0 | 0 | 0 | 0 | 0.064 | 0.061 | 0.125 | 0.06 | 0.06 | 0.12 |  |  |  |  |
| **11-15** | 0 | 0 | 0 | 0 | 0 | 0 | 0.097 | 0.093 | 0.190 | 0.10 | 0.09 | 0.19 |  |  |  |  |
| **16-20** | 2 | 4 | 6 | 54 | 111 | 166 | 0.127 | 0.116 | 0.244 | 54.59 | 111.28 | 165.87 | 276,426 | 8,228,411 | 56,835 | 658,156 |
| **21-25** | 4 | 6 | 10 | 105 | 162 | 267 | 0.231 | 0.186 | 0.417 | 105.46 | 161.78 | 267.24 | 48,020,545 | 497,206,288 | 23,940,807 | 146,475,468 |
| **26-30** | 18 | 17 | 35 | 454 | 441 | 896 | 0.301 | 0.243 | 0.545 | 454.69 | 441.60 | 896.29 |  |  |  |  |
| **31-35** | 51 | 14 | 65 | 1225 | 348 | 1573 | 0.300 | 0.236 | 0.536 | 1225.27 | 348.08 | 1573.35 | 452,617,170 | 634,166,652 | 62,820,542 | 175,302,837 |
| **35-40** | 92 | 32 | 124 | 2083 | 754 | 2837 | 0.325 | 0.256 | 0.580 | 2083.27 | 754.21 | 2837.47 |  |  |  |  |
| **40-45** | 137 | 32 | 169 | 2895 | 707 | 3602 | 0.329 | 0.259 | 0.587 | 2895.39 | 706.86 | 3602.25 | 1,045,436,941 | 579,131,146 | 84,708,658 | 127,953,283 |
| 45-50 | 171 | 49 | 220 | 3326 | 1000 | 4327 | 0.302 | 0.255 | 0.557 | 3326.56 | 1000.65 | 4327.21 |  |  |  |  |
| 50-55 | 255 | 86 | 341 | 4485 | 1599 | 6084 | 0.299 | 0.250 | 0.549 | 4485.18 | 1599.29 | 6084.47 | 997,108,023 | 438,672,390 | 119,541,254 | 100,779,910 |
| 55-60 | 276 | 133 | 409 | 4300 | 2210 | 6510 | 0.258 | 0.229 | 0.487 | 4300.41 | 2209.91 | 6510.32 |  |  |  |  |
| 60-65 | 306 | 170 | 476 | 4166 | 2481 | 6647 | 0.206 | 0.184 | 0.390 | 4166.34 | 2481.38 | 6647.72 |  |  |  |  |
| 65-70 | 305 | 175 | 480 | 3527 | 2169 | 5696 | 0.150 | 0.130 | 0.279 | 3526.80 | 2169.34 | 5696.14 |  |  |  |  |
| 70-75 | 326 | 188 | 514 | 3148 | 1947 | 5094 | 0.098 | 0.084 | 0.182 | 3147.65 | 1946.80 | 5094.44 |  |  |  |  |
| 75-80 | 202 | 147 | 349 | 1578 | 1226 | 2804 | 0.056 | 0.054 | 0.110 | 1577.86 | 1226.22 | 2804.08 |  |  |  |  |
| 80+ | 264 | 191 | 455 | 1624 | 1237 | 2861 | 0.561 | 0.517 | 1.078 | 1624.49 | 1237.34 | 2861.83 |  |  |  |  |
| **Total** | **2409** | **1244** | **3653** | **32970** | **16392** | **49362** | **4** | **3** | **7** | **32974** | **16395** | **49369** | **2,543,459,105** | **2,157,404,886** | **291,068,096** | **551,169,653** |

| Table S7: Age-specific Health and Economic Burden Indicators Associated with COVID-19- Gujarat | | | | | | | | | | | | | | | | | | | | | | | | |  |  | |  | |  | |  |  |  |
| --- | --- | --- | --- | --- | --- | --- | --- | --- | --- | --- | --- | --- | --- | --- | --- | --- | --- | --- | --- | --- | --- | --- | --- | --- | --- | --- | --- | --- | --- | --- | --- | --- | --- | --- |
| **Age group** | **Deaths** | | | | **YLL discounted** | | | | | | | | **YLD discounted** | | | | | | **DALYS** | | | | | | **CPL due to premature mortality** | **CPL due to absenteeism from work** | | **CPL due to premature mortality** | | **CPL due to absenteeism from work** | |  |  |  |
|  | **Male** | **Female** | **Both** | | **Male** | | **Female** | | **Both** | | **Male** | | | | **Female** | | **Both** | | **Male** | | **Female** | | **Both** | | **Male** | | | **Female** | | | |  |  |  |
| **<1** | 3 | 3 | 6 | | 99 | | 80 | | 179 | | 0.03 | | | | 0.02 | | 0.04 | | 99.03 | | 80.27 | | 179.30 | |  |  | |  | |  | |  |  |  |
| **1-9** | 10 | 5 | 15 | | 280 | | 161 | | 440 | | 0.20 | | | | 0.18 | | 0.38 | | 280.12 | | 160.69 | | 440.82 | |  | | | | | | |  |  |  |
| **10-19** | 21 | 18 | 39 | | 587 | | 511 | | 1098 | | 0.76 | | | | 0.63 | | 1.39 | | 587.60 | | 512.06 | | 1099.66 | | 4,996,158 | 85,398,576 | | 2,411,784 | | 31,455,564 | |  |  |  |
| **20-29** | 125 | 77 | 202 | | 3286 | | 2097 | | 5383 | | 2.47 | | | | 1.91 | | 4.38 | | 3288.67 | | 2099.11 | | 5387.79 | | 178,893,637 | 1,579,410,171 | | 38,423,901 | | 314,674,853 | |  |  |  |
| **30-39** | 434 | 170 | 604 | | 10378 | | 4288 | | 14666 | | 3.33 | | | | 2.30 | | 5.64 | | 10381.01 | | 4290.49 | | 14671.50 | | 817,446,402 | 2,144,763,383 | | 138,066,607 | | 484,501,338 | |  |  |  |
| **40-49** | 847 | 396 | 1243 | | 17811 | | 8924 | | 26735 | | 3.58 | | | | 2.56 | | 6.14 | | 17814.82 | | 8926.19 | | 26741.01 | | 1,506,085,505 | 1,781,747,178 | | 353,247,402 | | 534,812,298 | |  |  |  |
| **50-59** | 1384 | 736 | 2120 | | 24079 | | 14100 | | 38179 | | 3.54 | | | | 2.66 | | 6.20 | | 24082.31 | | 14103.15 | | 38185.45 | | 1,318,852,138 | 1,500,145,491 | | 261,119,950 | | 362,833,001 | |  |  |  |
| **60-69** | 1782 | 999 | 2781 | | 24055 | | 15351 | | 39406 | | 2.49 | | | | 1.85 | | 4.34 | | 24057.36 | | 15352.82 | | 39410.17 | |  |  | |  | |  | |  |  |  |
| **70-79** | 1391 | 699 | 2090 | | 13330 | | 6450 | | 19780 | | 1.00 | | | | 0.74 | | 1.74 | | 13330.75 | | 6451.22 | | 19781.97 | |  | | | | | | |  |  |  |
| **80+** | 666 | 328 | 994 | | 3821 | | 2410 | | 6231 | | 0.30 | | | | 0.24 | | 0.54 | | 3820.98 | | 2410.69 | | 6231.66 | |  | | | | | | |  |  |  |
| **Total** | **6663** | **3431** | **10094** | | **97725** | | **54374** | | **152099** | | **17.70** | | | | **13.08** | | **30.78** | | **97742.64** | | **54386.69** | | **152129.34** | | **3,826,273,840** | **7,091,464,799** | | **793,269,644** | | **1,728,277,056** | |  |  |  |
| Table S8: Age-specific Health and Economic Burden Indicators Associated with COVID-19- Haryana | | | | | | | | | | | | | | | | | | | |  | |  | |  | | |  | |  | |  | | | |
| **Age group** | **Deaths** | | | **YLL discounted** | | | | | | | | **YLD discounted** | | | | | | **DALYS** | | | | | | **CPL due to premature mortality** | | | **CPL due to absenteeism from work** | | **CPL due to premature mortality** | | **CPL due to absenteeism from work** | | | |
|  | **Male** | **Female** | **Both** | **Male** | | **Female** | | **Both** | | **Male** | | | | **Female** | | **Both** | | **Male** | | **Female** | | **Both** | | **Male** | | | | | **Female** | | | |  |  |
| **<1** | 3 | 3 | 6 | 96 | | 78 | | 175 | | 0.03 | | | | 0.02 | | 0.05 | | 96.53 | | 78.46 | | 174.99 | |  | | |  | |  | |  | | |  |
| **1-9** | 9 | 5 | 15 | 272 | | 157 | | 429 | | 0.36 | | | | 0.32 | | 0.68 | | 272.66 | | 157.17 | | 429.83 | |  | | | | | | | | |  |  |
| **10-19** | 20 | 17 | 38 | 570 | | 500 | | 1071 | | 0.97 | | | | 0.82 | | 1.79 | | 571.38 | | 501.11 | | 1072.49 | | 2,621,498 | | | 57,406,121 | | 386,418 | | 6,723,246 | | |  |
| **20-29** | 123 | 75 | 198 | 3188 | | 2054 | | 5242 | | 2.82 | | | | 2.19 | | 5.01 | | 3190.85 | | 2056.43 | | 5247.29 | | 167,226,350 | | | 1,715,620,350 | | 17,753,489 | | 170,408,212 | | |  |
| **30-39** | 424 | 166 | 590 | 10042 | | 4203 | | 14245 | | 3.77 | | | | 2.70 | | 6.47 | | 10046.17 | | 4205.50 | | 14251.67 | | 851,829,026 | | | 2,533,541,268 | | 106,838,438 | | 449,495,570 | | |  |
| **40-49** | 829 | 387 | 1216 | 17238 | | 8754 | | 25992 | | 3.17 | | | | 2.31 | | 5.48 | | 17240.87 | | 8756.43 | | 25997.30 | | 1,619,969,749 | | | 1,720,147,491 | | 224,790,704 | | 313,507,487 | | |  |
| **50-59** | 1354 | 720 | 2074 | 23489 | | 13854 | | 37343 | | 2.64 | | | | 2.17 | | 4.81 | | 23491.78 | | 13856.40 | | 37348.18 | | 1,424,953,722 | | | 1,186,499,140 | | 209,050,819 | | 243,041,351 | | |  |
| **60-69** | 1744 | 977 | 2721 | 23427 | | 14911 | | 38337 | | 1.85 | | | | 1.53 | | 3.37 | | 23428.52 | | 14912.25 | | 38340.77 | |  | | |  | |  | |  | | |  |
| **70-79** | 1361 | 684 | 2045 | 13520 | | 7729 | | 21249 | | 0.79 | | | | 0.61 | | 1.41 | | 13521.08 | | 7729.79 | | 21250.87 | |  | | | | | | | | |  |  |
| **80+** | 651 | 321 | 972 | 4579 | | 2482 | | 7061 | | 0.22 | | | | 0.17 | | 0.40 | | 4579.57 | | 2482.24 | | 7061.81 | |  | | | | | | | | |  |  |
| **Total** | **6518** | **3356** | **9874** | **96423** | | **54723** | | **151146** | | **16.61** | | | | **12.85** | | **29.46** | | **96439.40** | | **54735.80** | | **151175.20** | | **4,066,600,346** | | | **7,213,214,369** | | **558,819,868** | | **1,183,175,866** | | |  |

| Table S9: Age-specific Health and Economic Burden Indicators Associated with COVID-19-Himachal Pradesh | | | | | | | | | | | |  |  |  |  |  |
| --- | --- | --- | --- | --- | --- | --- | --- | --- | --- | --- | --- | --- | --- | --- | --- | --- |
| **Age group** | **Deaths** | | | **YLL discounted** | | | | **YLD** | | **DALYS** | | | **CPL due to premature mortality** | **CPL due to absenteeism from work** | **CPL due to premature mortality** | **CPL due to absenteeism from work** |
|  | **Male** | **Female** | **Both** | **Male** | **Female** | **Both** | **Male** | **Female** | **Both** | **Male** | **Female** | **Both** | **Male** |  | **Female** |  |
| <1 | 1 | 1 | 2 | 36 | 30 | 66 | 0.007 | 0.005 | 0.012 | 36.28 | 29.57 | 65.85 |  |  |  |  |
| 1-9 | 3 | 2 | 5 | 102 | 59 | 161 | 0.100 | 0.093 | 0.193 | 102.14 | 58.93 | 161.07 |  |  |  |  |
| 10-19 | 8 | 6 | 14 | 214 | 188 | 402 | 0.296 | 0.269 | 0.566 | 214.45 | 188.55 | 402.99 | 1163037.492 | 19243197.95 | 761464.0901 | 11742330.52 |
| 20-29 | 45 | 28 | 73 | 1203 | 777 | 1980 | 0.584 | 0.485 | 1.069 | 1203.61 | 777.61 | 1981.21 | 50119298.63 | 269772430.6 | 23748178.65 | 135702901.1 |
| 30-39 | 157 | 62 | 219 | 3814 | 1602 | 5416 | 0.782 | 0.599 | 1.381 | 3814.73 | 1602.56 | 5417.29 | 247459262.8 | 395084701.5 | 80991242.86 | 203690290.5 |
| 40-49 | 307 | 144 | 451 | 6564 | 3380 | 9944 | 0.772 | 0.612 | 1.384 | 6565.11 | 3380.76 | 9945.87 | 466224746.3 | 313300859.2 | 175628410.6 | 174855052.4 |
| 50-59 | 502 | 267 | 769 | 8951 | 5443 | 14394 | 0.609 | 0.503 | 1.112 | 8951.73 | 5443.31 | 14395.04 | 421346587.3 | 217931326.9 | 188930581.4 | 136999957.7 |
| 60-69 | 647 | 363 | 1009 | 8958 | 6041 | 14999 | 0.381 | 0.330 | 0.710 | 8958.51 | 6041.69 | 15000.20 |  |  |  |  |
| 70-79 | 505 | 254 | 758 | 5051 | 3240 | 8291 | 0.176 | 0.143 | 0.319 | 5051.23 | 3240.23 | 8291.46 |  |  |  |  |
| 80+ | 242 | 119 | 361 | 1680 | 1191 | 2871 | 0.058 | 0.051 | 0.109 | 1679.79 | 1191.49 | 2871.28 |  |  |  |  |
| **Total** | **2418** | **1245** | **3663** | **36574** | **21952** | **58525** | **4** | **3** | **7** | **36578** | **21955** | **58532** | **1,186,312,932** | **1,215,332,516** | **470,059,878** | **662,990,532** |

| Table S10: Age-specific Health and Economic Burden Indicators Associated with COVID-19-Jammu and Kashmir | | | | | | | | | | | |  |  |  | |  |  | | |
| --- | --- | --- | --- | --- | --- | --- | --- | --- | --- | --- | --- | --- | --- | --- | --- | --- | --- | --- | --- |
| **Age group** | **Deaths** | | | **YLL discounted** | | | | **YLD** | | **DALYS** | | | **CPL due to premature mortality** | **CPL due to absenteeism from work** | | **CPL due to premature mortality** | **CPL due to absenteeism from work** | | |
|  | **Male** | **Female** | **Both** | **Male** | **Female** | **Both** | **Male** | **Female** | **Both** | **Male** | **Female** | **Both** | **Male** | | **Female** | | |  |  |
| **<1** | 0 | 0 | 0 | 0 | 0 | 0 | 0.000 | 0.00 | 0.00 | 0.00 | 0.00 | 0.00 |  |  | |  |  | |  |
| **1-5** | 0 | 0 | 0 | 0 | 0 | 0 | 0.000 | 0.00 | 0.00 | 0.00 | 0.00 | 0.00 |  |  | |  |  | |  |
| **6-10** | 0 | 0 | 0 | 0 | 0 | 0 | 0.000 | 0.00 | 0.00 | 0.00 | 0.00 | 0.00 |  |  | |  |  | |  |
| **11-15** | 10 | 6 | 16 | 286 | 175 | 460 | 0.229 | 0.21 | 0.44 | 286.00 | 174.75 | 460.74 |  |  | |  |  | |  |
| **16-20** | 0 | 10 | 10 | 0 | 284 | 284 | 0.205 | 0.18 | 0.38 | 0.21 | 284.35 | 284.55 | 0 | 23,041,384 | | 622,627 | 4,353,573 | |  |
| **21-25** | 28 | 24 | 52 | 755 | 663 | 1418 | 0.402 | 0.31 | 0.71 | 755.03 | 663.63 | 1418.66 | 32,643,941 | 287,377,541 | | 4,582,822 | 27,145,782 | |  |
| **26-30** | 25 | 19 | 44 | 649 | 508 | 1157 | 0.633 | 0.47 | 1.10 | 649.69 | 508.47 | 1158.16 |  |  |  |  |  |  |  |
| **31-35** | 52 | 35 | 87 | 1291 | 900 | 2191 | 0.594 | 0.40 | 0.99 | 1291.27 | 900.42 | 2191.69 | 114,427,391 | 385,150,886 | | 23,970,776 | 56,128,611 | |  |
| **35-40** | 78 | 52 | 130 | 1838 | 1276 | 3114 | 0.621 | 0.38 | 1.00 | 1838.38 | 1276.20 | 3114.58 |  |  |  |  |  |  |  |
| **40-45** | 115 | 69 | 184 | 2543 | 1601 | 4144 | 0.552 | 0.33 | 0.88 | 2543.77 | 1601.32 | 4145.09 | 233,604,761 | 266,901,009 | | 60,479,864 | 50,227,228 | |  |
| **45-50** | 161 | 118 | 279 | 3312 | 2565 | 5877 | 0.490 | 0.32 | 0.81 | 3312.30 | 2565.47 | 5877.76 |  |  |  |  |  |  |  |
| **50-55** | 207 | 160 | 367 | 3912 | 3210 | 7122 | 0.441 | 0.31 | 0.75 | 3912.69 | 3210.33 | 7123.02 | 250,587,993 | 184,398,032 | | 71,466,829 | 42,870,400 | |  |
| **55-60** | 332 | 246 | 578 | 5680 | 4508 | 10188 | 0.437 | 0.33 | 0.76 | 5680.38 | 4508.45 | 10188.83 |  |  |  |  |  |  |  |
| **60-65** | 395 | 303 | 698 | 6005 | 5019 | 11024 | 0.329 | 0.24 | 0.57 | 6005.72 | 5019.10 | 11024.82 |  |  | |  |  | |  |
| **65-70** | 424 | 217 | 641 | 5697 | 3167 | 8864 | 0.233 | 0.16 | 0.39 | 5697.33 | 3167.33 | 8864.66 |  |  | |  |  | |  |
| **70-75** | 354 | 164 | 518 | 4093 | 2064 | 6157 | 0.126 | 0.08 | 0.21 | 4093.35 | 2063.71 | 6157.06 |  |  | |  |  | |  |
| **75-80** | 249 | 141 | 390 | 2422 | 1470 | 3892 | 0.084 | 0.06 | 0.14 | 2421.85 | 1469.80 | 3891.66 |  | | | | |  |  |
| **80+** | 295 | 137 | 432 | 2327 | 1112 | 3439 | 0.561 | 0.52 | 1.08 | 2327.33 | 1112.32 | 3439.65 |  | | | | |  |  |
| **Total** | **2725** | **1701** | **4426** | **40809** | **28521** | **69331** | **6** | **4** | **10** | **40815** | **28526** | **69341** | **631,264,086** | **1,146,868,851** | | **161,122,918** | **180,725,593** | |  |

| Table S11: Age-specific Health and Economic Burden Indicators Associated with COVID-19- Jharkhand | | | | | | | | | | | | | | |  |  |  | |
| --- | --- | --- | --- | --- | --- | --- | --- | --- | --- | --- | --- | --- | --- | --- | --- | --- | --- | --- |
| **Age group** | **Deaths** | | | **YLL discounted** | | | | **YLD discounted** | | | **DALYS** | | | **CPL due to premature mortality** | **CPL due to absenteeism from work** | **CPL due to premature mortality** | **CPL due to absenteeism from work** | |
|  | **Male** | **Female** | **Both** | **Male** | **Female** | **Both** | **Male** | | **Female** | **Both** | **Male** | **Female** | **Both** | **Male** | | **Female** | |  |
| **<1** | 2 | 1 | 3 | 51 | 40 | 91 | 0.02 | | 0.01 | 0.04 | 50.94 | 40.16 | 91.10 |  |  |  |  | |
| **1-9** | 5 | 3 | 8 | 143 | 80 | 224 | 0.13 | | 0.12 | 0.25 | 143.55 | 80.33 | 223.88 |  | | | |  |
| **10-19** | 11 | 9 | 20 | 302 | 254 | 556 | 0.47 | | 0.42 | 0.89 | 302.16 | 254.66 | 556.82 | 995,537 | 19,452,270 | 449,663 | 7,691,577 | |
| **20-29** | 64 | 39 | 103 | 1699 | 1034 | 2733 | 1.22 | | 0.93 | 2.16 | 1700.36 | 1034.69 | 2735.05 | 27,146,505 | 235,584,900 | 8,868,296 | 69,646,567 | |
| **30-39** | 221 | 87 | 307 | 5397 | 2086 | 7483 | 1.53 | | 0.93 | 2.46 | 5398.85 | 2087.00 | 7485.84 | 135,581,856 | 340,302,046 | 34,169,820 | 95,398,494 | |
| **40-49** | 431 | 201 | 633 | 9345 | 4258 | 13603 | 1.52 | | 0.92 | 2.44 | 9346.84 | 4258.65 | 13605.49 | 251,098,680 | 264,739,174 | 76,110,446 | 81,007,444 | |
| **50-59** | 704 | 375 | 1079 | 12783 | 6518 | 19301 | 1.28 | | 0.78 | 2.06 | 12783.88 | 6518.93 | 19302.81 | 230,429,759 | 199,950,111 | 61,396,054 | 49,226,057 | |
| **60-69** | 907 | 509 | 1416 | 12776 | 6680 | 19456 | 0.68 | | 0.46 | 1.14 | 12776.87 | 6680.29 | 19457.16 |  |  |  |  | |
| **70-79** | 708 | 356 | 1064 | 7035 | 3154 | 10189 | 0.26 | | 0.16 | 0.42 | 7035.63 | 3153.87 | 10189.51 |  | | | |  |
| **80+** | 339 | 167 | 506 | 2356 | 775 | 3131 | 0.07 | | 0.04 | 0.11 | 2356.19 | 775.40 | 3131.59 |  | | | |  |
| **Total** | **3392** | **1746** | **5138** | **51888** | **24879** | **76767** | **7.19** | | **4.78** | **11.97** | **51895.27** | **24883.98** | **76779.25** | **645,252,337** | **1,060,028,502** | **180,994,279** | **302,970,139** | |

| Table S12: Age-specific Health and Economic Burden Indicators Associated with COVID-19-Karnataka | | | | | | | | | | |  |  |  |  |  |  |
| --- | --- | --- | --- | --- | --- | --- | --- | --- | --- | --- | --- | --- | --- | --- | --- | --- |
| **Age group** | **Deaths** | | | **YLL discounted** | | | | **YLD** | | **DALYS** | | | **CPL due to premature mortality** | **CPL due to absenteeism from work** | **CPL due to premature mortality** | **CPL due to absenteeism from work** |
|  | **Male** | **Female** | **Both** | **Male** | **Female** | **Both** | **Male** | **Female** | **Both** | **Male** | **Female** | **Both** | **Male** | | **Female** | |
| **<1** | 0 | 0 | 0 | 0 | 0 | 0 | 0.142 | 0.09 | 0.23 | 0.14 | 0.09 | 0.23 |  |  |  |  |
| **1-5** | 0 | 0 | 0 | 0 | 0 | 0 | 0.668 | 0.63 | 1.29 | 0.67 | 0.63 | 1.29 |  |  |  |  |
| **6-10** | 0 | 0 | 0 | 0 | 0 | 0 | 0.795 | 0.77 | 1.56 | 0.79 | 0.77 | 1.56 |  |  |  |  |
| **11-15** | 0 | 0 | 0 | 0 | 0 | 0 | 1.391 | 1.32 | 2.71 | 1.39 | 1.32 | 2.71 |  |  |  |  |
| **16-20** | 76 | 60 | 136 | 2050 | 1663 | 3713 | 2.402 | 2.24 | 4.64 | 2052.12 | 1665.64 | 3717.76 | 23,610,525 | 337,694,991 | 4,906,911 | 72,871,997 |
| **21-25** | 102 | 117 | 219 | 2650 | 3142 | 5792 | 4.357 | 3.89 | 8.25 | 2654.76 | 3145.85 | 5800.62 | 640,858,977 | 6,223,982,454 | 165,429,153 | 1,561,926,817 |
| **26-30** | 317 | 185 | 502 | 7892 | 4782 | 12675 | 5.818 | 4.81 | 10.63 | 7898.14 | 4787.23 | 12685.37 |  |  |  |  |
| **31-35** | 619 | 224 | 843 | 14638 | 5537 | 20175 | 5.892 | 4.54 | 10.43 | 14644.33 | 5541.34 | 20185.68 | 1,968,943,370 | 8,447,451,973 | 452,120,295 | 2,712,373,858 |
| **35-40** | 1054 | 432 | 1486 | 23450 | 10115 | 33565 | 6.413 | 4.85 | 11.26 | 23456.56 | 10119.39 | 33575.95 |  |  |  |  |
| **40-45** | 1417 | 638 | 2055 | 29256 | 13979 | 43236 | 6.151 | 4.51 | 10.66 | 29262.36 | 13983.82 | 43246.18 | 3,305,036,000 | 6,310,066,021 | 713,436,982 | 2,240,753,322 |
| **45-50** | 1923 | 1062 | 2985 | 36510 | 21475 | 57985 | 5.258 | 4.14 | 9.40 | 36515.51 | 21478.78 | 57994.29 |  |  |  |  |
| **50-55** | 2254 | 1328 | 3582 | 38562 | 24337 | 62899 | 4.812 | 3.81 | 8.62 | 38566.83 | 24340.36 | 62907.19 | 2,618,085,318 | 4,234,729,307 | 501,506,780 | 1,377,152,113 |
| **55-60** | 2776 | 1710 | 4486 | 41750 | 27891 | 69641 | 4.507 | 3.81 | 8.32 | 41754.47 | 27894.70 | 69649.17 |  |  |  |  |
| **60-65** | 3093 | 1886 | 4979 | 40253 | 26993 | 67246 | 3.707 | 3.06 | 6.77 | 40256.95 | 26995.61 | 67252.56 |  |  |  |  |
| **65-70** | 3169 | 1975 | 5144 | 34967 | 23856 | 58823 | 2.559 | 2.00 | 4.56 | 34969.23 | 23858.14 | 58827.37 |  |  |  |  |
| **70-75** | 2960 | 1594 | 4554 | 26227 | 15840 | 42068 | 1.587 | 1.17 | 2.76 | 26229.08 | 15841.55 | 42070.63 |  |  |  |  |
| **75-80** | 1972 | 1079 | 3051 | 14948 | 8919 | 23867 | 0.948 | 0.72 | 1.67 | 14949.02 | 8919.94 | 23868.96 |  | | | |
| **80+** | 2456 | 1299 | 3755 | 14302 | 7990 | 22292 | 0.561 | 0.52 | 1.08 | 14302.11 | 7991.00 | 22293.10 |  | | | |
| **Total** | **24188** | **13589** | **37777** | **327456** | **196519** | **523976** | **58** | **47** | **105** | **327514** | **196566** | **524081** | **8,556,534,191** | **25,553,924,746** | **1,837,400,121** | **7,965,078,106** |

| Table S13: Age-specific Health and Economic Burden Indicators Associated with COVID-19-Kerala | | | | | | | | | | |  |  |  |  |  |  |
| --- | --- | --- | --- | --- | --- | --- | --- | --- | --- | --- | --- | --- | --- | --- | --- | --- |
| **Age group** | **Deaths** | | | **YLL discounted** | | | | **YLD** | | **DALYS** | | | **CPL due to premature mortality** | **CPL due to absenteeism from work** | **CPL due to premature mortality** | **CPL due to absenteeism from work** |
|  | **Male** | **Female** | **Both** | **Male** | **Female** | **Both** | **Male** | **Female** | **Both** | **Male** | **Female** | **Both** | **Male** | | **Female** | |
| **<1** | 10 | 10 | 20 | 0 | 0 | 0 | 0.030 | 0.02 | 0.05 | 0.03 | 0.02 | 0.05 |  |  |  |  |
| **1-5** | 4 | 4 | 8 | 0 | 0 | 0 | 1.740 | 1.67 | 3.41 | 1.74 | 1.67 | 3.41 |  |  |  |  |
| **6-10** | 6 | 4 | 10 | 0 | 0 | 0 | 1.665 | 1.62 | 3.29 | 1.66 | 1.62 | 3.29 |  |  |  |  |
| **11-15** | 12 | 8 | 20 | 0 | 0 | 0 | 2.367 | 2.28 | 4.65 | 2.37 | 2.28 | 4.65 |  |  |  |  |
| **16-20** | 16 | 20 | 36 | 440 | 569 | 1009 | 2.941 | 2.86 | 5.80 | 442.66 | 571.79 | 1014.45 | 6,983,392 | 539,418,632 | 2,483,980 | 333,887,887 |
| **21-25** | 42 | 30 | 72 | 1115 | 830 | 1946 | 4.152 | 4.01 | 8.16 | 1119.51 | 834.19 | 1953.70 | 121,633,134 | 3,781,540,080 | 19,558,806 | 2,077,880,043 |
| **26-30** | 57 | 62 | 119 | 1454 | 1660 | 3114 | 4.531 | 4.23 | 8.76 | 1458.40 | 1664.35 | 3122.75 |  |  |  |  |
| **31-35** | 134 | 70 | 204 | 3259 | 1802 | 5061 | 4.747 | 4.36 | 9.11 | 3263.80 | 1806.01 | 5069.81 | 746,217,730 | 5,108,805,697 | 136,852,322 | 3,095,031,412 |
| **35-40** | 291 | 179 | 470 | 6680 | 4396 | 11077 | 5.634 | 5.30 | 10.93 | 6686.03 | 4401.79 | 11087.82 |  |  |  |  |
| **40-45** | 397 | 223 | 620 | 8490 | 5174 | 13664 | 5.563 | 5.16 | 10.72 | 8495.57 | 5179.37 | 13674.94 | 1,845,367,037 | 4,738,424,294 | 417,410,598 | 3,195,277,494 |
| **45-50** | 696 | 424 | 1120 | 13682 | 9188 | 22870 | 5.786 | 5.59 | 11.38 | 13688.09 | 9193.14 | 22881.23 |  |  |  |  |
| **50-55** | 974 | 643 | 1617 | 17313 | 12797 | 30111 | 5.439 | 5.14 | 10.58 | 17318.87 | 12802.41 | 30121.28 | 2,202,546,555 | 3,804,250,540 | 574,449,382 | 2,617,929,281 |
| **55-60** | 1401 | 993 | 2394 | 22051 | 17835 | 39886 | 4.905 | 4.73 | 9.63 | 22055.71 | 17840.17 | 39895.88 |  |  |  |  |
| **60-65** | 2043 | 1279 | 3322 | 27694 | 20265 | 47959 | 4.230 | 3.95 | 8.18 | 27698.27 | 20269.17 | 47967.45 |  |  |  |  |
| **65-70** | 2075 | 1388 | 3463 | 23584 | 18897 | 42482 | 2.887 | 2.66 | 5.54 | 23587.27 | 18900.03 | 42487.30 |  |  |  |  |
| **70-75** | 2328 | 1526 | 3854 | 21979 | 17445 | 39424 | 1.896 | 1.77 | 3.67 | 21980.53 | 17446.66 | 39427.19 |  |  |  |  |
| **75-80** | 1652 | 1271 | 2923 | 12266 | 11725 | 23991 | 1.112 | 1.15 | 2.26 | 12267.50 | 11726.11 | 23993.61 |  | | | |
| **80+** | 2342 | 2349 | 4691 | 13250 | 16522 | 29772 | 0.561 | 0.52 | 1.08 | 13250.54 | 16522.50 | 29773.04 |  | | | |
| **Total** | **14480** | **10483** | **24963** | **173258** | **139106** | **312365** | **60** | **57** | **117** | **173319** | **139163** | **312482** | **4,922,747,847** | **17,972,439,244** | **1,150,755,088** | **11,320,006,117** |

| Table S14: Age-specific Health and Economic Burden Indicators Associated with COVID-19-Maharashtra | | | | | | | | | | | | | |  |  |  |  |
| --- | --- | --- | --- | --- | --- | --- | --- | --- | --- | --- | --- | --- | --- | --- | --- | --- | --- |
| **Age group** | **Deaths** | | | **YLL discounted** | | | | **YLD discounted** | | | **DALYS** | | | **CPL due to premature mortality** | **CPL due to absenteeism from work** | **CPL due to premature mortality** | **CPL due to absenteeism from work** |
|  | **Male** | **Female** | **Both** | **Male** | **Female** | **Both** | **Male** | | **Female** | **Both** | **Male** | **Female** | **Both** | **Male** | | **Female** | |
| **<1** | 47 | 37 | 84 | 1384 | 1110 | 2493 | 12.625 | | 8.86 | 21.48 | 1396.37 | 1118.58 | 2514.95 |  |  |  |  |
| **1-9** | 132 | 74 | 206 | 3883 | 2208 | 6091 | 2.853 | | 2.65 | 5.51 | 3886.10 | 2210.87 | 6096.97 |  | | | |
| **10-19** | 288 | 245 | 533 | 8158 | 7030 | 15188 | 6.353 | | 5.68 | 12.03 | 8164.13 | 7035.55 | 15199.68 | 32,551,895 | 310,549,606 | 14,326,981 | 122,800,829 |
| **20-29** | 1726 | 1061 | 2786 | 45936 | 28823 | 74760 | 15.575 | | 13.29 | 28.86 | 45951.91 | 28836.66 | 74788.56 | 1,898,184,662 | 6,967,936,434 | 446,333,700 | 1,842,439,823 |
| **30-39** | 5971 | 2342 | 8313 | 145708 | 58826 | 204534 | 22.042 | | 16.72 | 38.76 | 145730.03 | 58842.81 | 204572.83 | 10,191,152,405 | 12,156,491,822 | 2,037,748,552 | 3,769,610,052 |
| **40-49** | 11669 | 5451 | 17120 | 252023 | 122004 | 374027 | 21.966 | | 16.66 | 38.62 | 252044.89 | 122020.77 | 374065.66 | 18,763,403,461 | 9,657,773,416 | 4,998,209,029 | 3,568,753,317 |
| **50-59** | 19060 | 10136 | 29197 | 346706 | 192452 | 539159 | 19.616 | | 15.46 | 35.08 | 346726.06 | 192467.69 | 539193.75 | 16,354,163,425 | 7,327,985,688 | 4,157,906,682 | 2,443,030,704 |
| **60-69** | 24546 | 13758 | 38304 | 352701 | 206162 | 558863 | 13.756 | | 10.85 | 24.61 | 352714.61 | 206172.90 | 558887.52 |  |  |  |  |
| **70-79** | 19154 | 9630 | 28784 | 198339 | 102353 | 300693 | 6.123 | | 4.58 | 10.70 | 198345.50 | 102358.05 | 300703.55 |  | | | |
| **80+** | 9166 | 4518 | 13684 | 63020 | 28892 | 91912 | 1.819 | | 1.37 | 3.19 | 63021.34 | 28893.56 | 91914.90 |  | | | |
| **Total** | **91759** | **47252** | **139011** | **1417858** | **749861** | **2167720** | **122.729** | | **96.11** | **218.84** | **1417980.94** | **749957.44** | **2167938.38** | **47,239,455,849** | **36,420,736,967** | **11,654,524,943** | **11,746,634,725** |

| Table S15: Age-specific Health and Economic Burden Indicators Associated with COVID-19-Madhya Pradesh | | | | | | | | | | |  |  |  |  |  |  |
| --- | --- | --- | --- | --- | --- | --- | --- | --- | --- | --- | --- | --- | --- | --- | --- | --- |
| **Age group** | **Deaths** | | | **YLL discounted** | | | | **YLD** | | **DALYS** | | | **CPL due to premature mortality** | **CPL due to absenteeism from work** | **CPL due to premature mortality** | **CPL due to absenteeism from work** |
|  | **Male** | **Female** | **Both** | **Male** | **Female** | **Both** | **Male** | **Female** | **Both** | **Male** | **Female** | **Both** | **Male** | | **Female** | |
| **<1** | 5 | 2 | 7 | 143 | 58 | 202 | 0.074 | 0.05 | 0.12 | 143.38 | 58.42 | 201.81 |  |  |  |  |
| **1-4** | 0 | 0 | 0 | 0 | 0 | 0 | 0.312 | 0.31 | 0.62 | 0.31 | 0.31 | 0.62 |  |  |  |  |
| **5-14** | 5 | 2 | 6 | 141 | 58 | 199 | 1.241 | 1.23 | 2.47 | 142.31 | 58.77 | 201.08 |  |  |  |  |
| **15-24** | 46 | 33 | 80 | 1220 | 900 | 2120 | 4.367 | 4.21 | 8.58 | 1224.55 | 903.76 | 2128.30 | 17970739.16 | 336523265.2 | 4631388.431 | 78668491.39 |
| **25-34** | 393 | 186 | 580 | 9585 | 4704 | 14289 | 6.138 | 5.97 | 12.11 | 9590.88 | 4710.34 | 14301.22 | 347673150.8 | 1134378600 | 58567789.16 | 226155569.2 |
| **35-44** | 1047 | 401 | 1448 | 22632 | 9098 | 31730 | 4.817 | 4.67 | 9.49 | 22636.91 | 9102.83 | 31739.74 | 976927899.1 | 938056135.3 | 189187565.4 | 259635957 |
| **45-54** | 1478 | 787 | 2265 | 26918 | 15193 | 42112 | 4.437 | 4.28 | 8.72 | 26922.86 | 15197.55 | 42120.41 | 1076473302 | 749072313.2 | 293602709.3 | 240001945.3 |
| **55-64** | 1765 | 957 | 2722 | 25008 | 14602 | 39610 | 3.469 | 3.33 | 6.80 | 25011.52 | 14605.07 | 39616.58 | 43855228.02 | 568465345.9 | 7815206.623 | 115516450.6 |
| **65-69** | 777 | 377 | 1154 | 8625 | 4506 | 13131 | 0.899 | 0.85 | 1.75 | 8626.20 | 4506.43 | 13132.63 |  |  |  |  |
| **70+** | 1532 | 727 | 2258 | 9828 | 5085 | 14913 | 1.210 | 1.12 | 2.33 | 9828.98 | 5085.86 | 14914.84 |  |  |  |  |
| **Total** | **7048** | **3472** | **10520** | **104101** | **54203** | **158304** | **27** | **26** | **53** | **104128** | **54229** | **158357** | **2,462,900,319** | **3,726,495,660** | **553,804,659** | **919,978,414** |

| Table S16: Age-specific Health and Economic Burden Indicators Associated with COVID-19-Odisha | | | | | | | | | | | |  |  |  |  |  |  |
| --- | --- | --- | --- | --- | --- | --- | --- | --- | --- | --- | --- | --- | --- | --- | --- | --- | --- |
| **Age group** | **Deaths** | | | **YLL discounted** | | | | **YLD discounted** | | | **DALYS** | | | **CPL due to premature mortality** | **CPL due to absenteeism from work** | **CPL due to premature mortality** | **CPL due to absenteeism from work** |
|  | **Male** | **Female** | **Both** | **Male** | **Female** | **Both** | **Male** | | **Female** | **Both** | **Male** | **Female** | **Both** | **Male** | | **Female** | |
| **<1** | 3 | 2 | 5 | 81 | 65 | 146 | 0.028 | | 0.02 | 0.05 | 80.79 | 65.15 | 145.94 |  |  |  |  |
| **1-9** | 8 | 4 | 12 | 228 | 130 | 358 | 0.355 | | 0.32 | 0.68 | 228.54 | 130.55 | 359.09 |  | | | |
| **10-19** | 17 | 14 | 31 | 480 | 415 | 895 | 0.970 | | 0.82 | 1.79 | 480.51 | 416.19 | 896.70 | 1,370,693 | 36,152,140 | 475,322 | 9,960,827 |
| **20-29** | 102 | 63 | 164 | 2699 | 1706 | 4404 | 2.819 | | 2.19 | 5.01 | 2701.53 | 1707.75 | 4409.28 | 61,712,085 | 762,556,291 | 14,590,516 | 168,679,641 |
| **30-39** | 352 | 138 | 490 | 8574 | 3489 | 12063 | 3.772 | | 2.70 | 6.47 | 8577.79 | 3492.13 | 12069.92 | 309,511,709 | 1,108,760,743 | 53,994,689 | 273,611,368 |
| **40-49** | 688 | 321 | 1010 | 14863 | 7268 | 22131 | 3.165 | | 2.31 | 5.48 | 14865.90 | 7270.52 | 22136.42 | 580,205,564 | 742,037,545 | 126,566,499 | 212,605,034 |
| **50-59** | 1124 | 598 | 1722 | 20548 | 11503 | 32051 | 2.640 | | 2.17 | 4.81 | 20551.04 | 11504.80 | 32055.84 | 531,145,551 | 532,678,815 | 119,356,530 | 167,132,031 |
| **60-69** | 1448 | 811 | 2259 | 20964 | 12380 | 33344 | 1.847 | | 1.53 | 3.37 | 20966.16 | 12381.32 | 33347.48 |  |  |  |  |
| **70-79** | 1130 | 568 | 1697 | 12312 | 6417 | 18730 | 0.791 | | 0.61 | 1.41 | 12313.15 | 6417.85 | 18731.00 |  | | | |
| **80+** | 541 | 266 | 807 | 4549 | 2061 | 6610 | 0.222 | | 0.17 | 0.40 | 4549.60 | 2060.94 | 6610.54 |  | | | |
| **Total** | **5411** | **2787** | **8198** | **85298** | **45434** | **130733** | **16.610** | | **12.85** | **29.46** | **85315.01** | **45447.20** | **130762.21** | **1,483,945,603** | **3,182,185,533** | **314,983,556** | **831,988,900** |

| Table S17: Age-specific Health and Economic Burden Indicators Associated with COVID-19-Punjab | | | | | | | | | | | | |  |  |  |  | |
| --- | --- | --- | --- | --- | --- | --- | --- | --- | --- | --- | --- | --- | --- | --- | --- | --- | --- |
| **Age group** | **Deaths** | | | **YLL discounted** | | | | **YLD** | | **DALYS** | | | **CPL due to premature mortality** | **CPL due to absenteeism from work** | **CPL due to premature mortality** | **CPL due to absenteeism from work** | |
|  | **Male** | **Female** | **Both** | **Male** | **Female** | **Both** | **Male** | **Female** | **Both** | **Male** | **Female** | **Both** | **Male** | | **Female** | |  |
| **<1** | 6 | 4 | 10 | 164 | 129 | 293 | 0.016 | 0.01 | 0.03 | 163.89 | 129.06 | 292.95 |  |  |  |  | |
| **1-9** | 16 | 9 | 24 | 460 | 258 | 718 | 0.179 | 0.16 | 0.34 | 460.45 | 258.00 | 718.46 |  | | | |  |
| **10-19** | 34 | 29 | 63 | 967 | 817 | 1784 | 0.657 | 0.52 | 1.18 | 967.28 | 817.78 | 1785.06 | 3,132,516 | 29,712,570 | 523,583 | 3,458,638 | |
| **20-29** | 205 | 126 | 331 | 5433 | 3323 | 8756 | 1.850 | 1.41 | 3.26 | 5434.57 | 3324.39 | 8758.95 | 188,888,419 | 776,278,782 | 26,219,776 | 96,429,567 | |
| **30-39** | 709 | 278 | 988 | 17235 | 6706 | 23941 | 2.536 | 1.76 | 4.30 | 17237.72 | 6707.37 | 23945.09 | 929,247,808 | 1,129,004,091 | 118,816,101 | 195,092,270 | |
| **40-49** | 1386 | 648 | 2034 | 29943 | 13686 | 43629 | 2.553 | 1.82 | 4.37 | 29945.58 | 13688.22 | 43633.80 | 1,720,151,630 | 890,744,462 | 265,236,578 | 173,718,660 | |
| **50-59** | 2265 | 1204 | 3469 | 41905 | 20952 | 62858 | 2.424 | 1.91 | 4.33 | 41907.52 | 20954.36 | 62861.88 | 1,469,095,297 | 685,184,678 | 232,045,481 | 141,524,236 | |
| **60-69** | 2916 | 1635 | 4551 | 44179 | 21472 | 65652 | 1.739 | 1.36 | 3.10 | 44181.13 | 21473.54 | 65654.67 |  |  |  |  | |
| **70-79** | 2276 | 1144 | 3420 | 27489 | 10138 | 37626 | 0.774 | 0.56 | 1.33 | 27489.44 | 10138.11 | 37627.55 |  | | | |  |
| **80+** | 1089 | 537 | 1626 | 10125 | 2492 | 12617 | 0.198 | 0.14 | 0.34 | 10125.05 | 2492.52 | 12617.57 |  | | | |  |
| **Total** | **10902** | **5614** | **16516** | **177900** | **79974** | **257873** | **13** | **10** | **23** | **177913** | **79983** | **257896** | **4,310,515,670** | **3,510,924,583** | **642,841,519** | **610,223,372** | |

| Table S18: Age-specific Health and Economic Burden Indicators Associated with COVID-19-Rajasthan | | | | | | | | | | |  |  |  |  |  |  |
| --- | --- | --- | --- | --- | --- | --- | --- | --- | --- | --- | --- | --- | --- | --- | --- | --- |
| **Age group** | **Deaths** | | | **YLL discounted** | | | |  | | **DALYS** | | | **CPL due to premature mortality** | **CPL due to absenteeism from work** | **CPL due to premature mortality** | **CPL due to absenteeism from work** |
|  | **Male** | **Female** | **Both** | **Male** | **Female** | **Both** | **Male** | **Female** | **Both** | **Male** | **Female** | **Both** | **Male** | | **Female** | |
| **<1** | 3 | 2 | 5 | 87 | 71 | 158 | 0.388 | 0.02 | 0.40 | 87.82 | 70.80 | 158.61 |  |  |  |  |
| **1-9** | 8 | 5 | 13 | 247 | 142 | 389 | 1.222 | 0.32 | 1.54 | 248.47 | 142.45 | 390.93 |  | | | |
| **10-19** | 19 | 16 | 34 | 518 | 453 | 971 | 2.259 | 1.03 | 3.29 | 520.44 | 454.06 | 974.49 | 1,547,990 | 130,803,476 | 597,076 | 14,431,646 |
| **20-29** | 111 | 68 | 179 | 2901 | 1859 | 4760 | 3.365 | 2.62 | 5.99 | 2904.07 | 1861.72 | 4765.79 | 74,641,071 | 1,005,975,989 | 22,612,864 | 286,255,745 |
| **30-39** | 385 | 151 | 535 | 9140 | 3800 | 12940 | 3.550 | 2.79 | 6.34 | 9143.53 | 3803.01 | 12946.53 | 391,813,335 | 1,141,299,965 | 85,941,612 | 412,455,568 |
| **40-49** | 752 | 351 | 1103 | 15575 | 7916 | 23491 | 3.338 | 2.66 | 6.00 | 15578.24 | 7918.46 | 23496.70 | 730,088,019 | 866,630,223 | 200,873,111 | 355,149,296 |
| **50-59** | 1228 | 653 | 1881 | 20944 | 12480 | 33424 | 2.687 | 2.44 | 5.12 | 20946.84 | 12482.63 | 33429.47 | 662,126,795 | 590,317,927 | 222,076,187 | 319,106,158 |
| **60-69** | 1581 | 886 | 2467 | 20864 | 13569 | 34434 | 1.632 | 1.57 | 3.20 | 20865.78 | 13570.99 | 34436.77 |  |  |  |  |
| **70-79** | 1234 | 620 | 1854 | 11648 | 6885 | 18533 | 0.582 | 0.57 | 1.15 | 11648.56 | 6886.03 | 18534.59 |  | | | |
| **80+** | 590 | 291 | 881 | 3535 | 1931 | 5466 | 0.162 | 0.17 | 0.33 | 3535.30 | 1931.42 | 5466.72 |  | | | |
| **Total** | **5910** | **3044** | **8954** | **85460** | **49107** | **134567** | **19** | **14** | **33** | **85479** | **49122** | **134601** | **1,860,217,210** | **3,735,027,581** | **532,100,850** | **1,387,398,413** |

| Table S19: Age-specific Health and Economic Burden Indicators Associated with COVID-19-Tamil Nadu | | | | | | | | | | |  |  |  |  |  |  |
| --- | --- | --- | --- | --- | --- | --- | --- | --- | --- | --- | --- | --- | --- | --- | --- | --- |
| **Age group** | **Deaths** | | | **YLL discounted** | | | | **YLD** | | **DALYS** | | | **CPL due to premature mortality** | **CPL due to absenteeism from work** | **CPL due to premature mortality** | **CPL due to absenteeism from work** |
|  | **Male** | **Female** | **Both** | **Male** | **Female** | **Both** | **Male** | **Female** | **Both** | **Male** | **Female** | **Both** | **Male** | | **Female** | |
| <=15 | 9 | 13 | 22 | 258 | 356 | 614 | 6.115 | 5.37 | 11.49 | 264.32 | 361.23 | 625.54 |  |  |  |  |
| 16-20 | 18 | 17 | 35 | 493 | 456 | 949 | 4.393 | 4.36 | 8.75 | 497.46 | 459.92 | 957.38 | 4,020,916 | 168,026,296 | 959,144 | 36,098,421 |
| 21-25 | 68 | 78 | 146 | 1765 | 2077 | 3842 | 7.958 | 7.87 | 15.83 | 1772.86 | 2085.34 | 3858.20 | 382,539,988 | 3,932,857,020 | 114,651,428 | 1,084,089,199 |
| 26-30 | 223 | 150 | 374 | 5568 | 3824 | 9392 | 9.385 | 9.12 | 18.50 | 5577.52 | 3832.86 | 9410.39 |  |  |  |  |
| 31-35 | 472 | 189 | 661 | 11185 | 4594 | 15778 | 8.407 | 8.07 | 16.47 | 11193.20 | 4601.62 | 15794.83 | 2,564,791,761 | 5,942,584,401 | 633,014,230 | 2,202,929,228 |
| 35-40 | 863 | 380 | 1243 | 19222 | 8727 | 27949 | 9.101 | 8.72 | 17.82 | 19231.36 | 8735.93 | 27967.29 |  |  |  |  |
| 40-45 | 1123 | 570 | 1693 | 23221 | 12236 | 35457 | 7.987 | 7.60 | 15.59 | 23229.40 | 12243.17 | 35472.57 | 5,026,244,154 | 5,106,486,660 | 1,629,441,285 | 2,293,859,192 |
| 45-50 | 1599 | 853 | 2452 | 30428 | 16880 | 47308 | 8.395 | 8.16 | 16.56 | 30436.31 | 16888.49 | 47324.80 |  |  |  |  |
| 50-55 | 2299 | 1339 | 3638 | 39445 | 23930 | 63375 | 8.168 | 7.86 | 16.03 | 39453.39 | 23937.40 | 63390.78 | 5,268,169,211 | 4,312,259,491 | 1,748,218,076 | 1,874,534,830 |
| 55-60 | 2991 | 1737 | 4728 | 45472 | 27698 | 73170 | 7.390 | 7.10 | 14.49 | 45479.50 | 27704.64 | 73184.14 |  |  |  |  |
| 60-65 | 3530 | 1876 | 5406 | 46797 | 26095 | 72892 | 5.551 | 5.26 | 10.81 | 46802.69 | 26099.77 | 72902.46 |  |  |  |  |
| 65-70 | 3349 | 1675 | 5024 | 37843 | 19916 | 57759 | 3.825 | 3.59 | 7.42 | 37846.62 | 19919.64 | 57766.25 |  |  |  |  |
| 70-75 | 3085 | 1329 | 4414 | 29346 | 13016 | 42361 | 2.426 | 2.24 | 4.67 | 29348.40 | 13017.75 | 42366.15 |  |  |  |  |
| 75-80 | 1961 | 840 | 2801 | 15018 | 6815 | 21833 | 1.240 | 1.15 | 2.39 | 15019.71 | 6815.85 | 21835.56 |  | | | |
| 80+ | 2160 | 782 | 2942 | 12759 | 4871 | 17630 | 0.561 | 0.52 | 1.08 | 12759.29 | 4871.85 | 17631.15 |  | | | |
| **Total** | **23751** | **11828** | **35579** | **318821** | **171488** | **490310** | **91** | **87** | **178** | **318912** | **171575** | **490487** | **13,245,766,029** | **19,462,213,869** | **4,126,284,163** | **7,491,510,871** |

| Table 20 Age-specific Health and Economic Burden Indicators Associated with COVID-19-Telangana | | | | | | | | | | | |  |  |  | |  |  | |
| --- | --- | --- | --- | --- | --- | --- | --- | --- | --- | --- | --- | --- | --- | --- | --- | --- | --- | --- |
| **Age group** | **Deaths** | | | **YLL discounted** | | | | **YLD** | | **DALYS** | | | **CPL due to premature mortality** | **CPL due to absenteeism from work** | | **CPL due to premature mortality** | **CPL due to absenteeism from work** | |
|  | **Male** | **Female** | **Both** | **Male** | **Female** | **Both** | **Male** | **Female** | **Both** | **Male** | **Female** | **Both** | **Male** | | **Female** | | |  |
| **<1** | 0 | 0 | 0 | 0 | 0 | 0 | 0.022 | 0.00 | 0.03 | 0.02 | 0.00 | 0.03 |  |  | |  |  | |
| **1-5** | 0 | 0 | 0 | 0 | 0 | 0 | 0.039 | 0.03 | 0.07 | 0.04 | 0.03 | 0.07 |  |  | |  |  | |
| **6-10** | 0 | 0 | 0 | 0 | 0 | 0 | 0.043 | 0.03 | 0.07 | 0.04 | 0.03 | 0.07 |  |  | |  |  | |
| **11-15** | 0 | 0 | 0 | 0 | 0 | 0 | 0.069 | 0.04 | 0.11 | 0.07 | 0.04 | 0.11 |  |  | |  |  | |
| **16-20** | 15 | 13 | 28 | 409 | 361 | 769 | 0.076 | 0.05 | 0.13 | 408.83 | 360.67 | 769.50 | 2,434,296 | 7,880,065 | | 1,212,304 | 1,981,102 | |
| **21-25** | 30 | 40 | 70 | 789 | 1075 | 1864 | 0.147 | 0.12 | 0.27 | 789.38 | 1075.08 | 1864.46 | 145,000,312 | 209,744,574 | | 37,553,223 | 55,701,037 | |
| **26-30** | 77 | 28 | 105 | 1942 | 724 | 2666 | 0.227 | 0.18 | 0.41 | 1942.14 | 724.64 | 2666.77 |  |  |  |  |  |  |
| **31-35** | 87 | 48 | 135 | 2090 | 1188 | 3277 | 0.233 | 0.17 | 0.40 | 2089.88 | 1187.86 | 3277.75 | 499,435,413 | 338,367,020 | | 174,006,785 | 126,643,827 | |
| **35-40** | 157 | 76 | 233 | 3555 | 1782 | 5336 | 0.252 | 0.18 | 0.43 | 3554.84 | 1781.85 | 5336.68 |  |  |  |  |  |  |
| **40-45** | 204 | 67 | 271 | 4311 | 1470 | 5781 | 0.245 | 0.17 | 0.42 | 4311.14 | 1470.51 | 5781.65 | 990,208,887 | 267,788,426 | | 248,488,623 | 114,200,560 | |
| **45-50** | 308 | 116 | 424 | 5991 | 2355 | 8346 | 0.240 | 0.18 | 0.42 | 5991.40 | 2354.91 | 8346.31 |  |  |  |  |  |  |
| **50-55** | 464 | 162 | 626 | 8204 | 2998 | 11202 | 0.233 | 0.18 | 0.41 | 8204.67 | 2997.95 | 11202.62 | 1,063,295,192 | 197,333,237 | | 243,180,259 | 82,337,965 | |
| **55-60** | 559 | 229 | 788 | 8857 | 3782 | 12639 | 0.203 | 0.16 | 0.36 | 8857.32 | 3781.76 | 12639.08 |  |  |  |  |  |  |
| **60-65** | 462 | 229 | 691 | 6426 | 3316 | 9742 | 0.148 | 0.12 | 0.27 | 6425.82 | 3316.61 | 9742.44 |  |  | |  |  | |
| **65-70** | 416 | 197 | 613 | 4998 | 2454 | 7453 | 0.103 | 0.08 | 0.19 | 4998.43 | 2454.35 | 7452.78 |  |  | |  |  | |
| **70-75** | 335 | 147 | 482 | 3353 | 1542 | 4895 | 0.062 | 0.05 | 0.11 | 3352.60 | 1542.42 | 4895.02 |  |  | |  |  | |
| **75-80** | 181 | 88 | 269 | 1483 | 773 | 2256 | 0.034 | 0.03 | 0.06 | 1482.58 | 773.30 | 2255.88 |  | | | | |  |
| **80+** | 204 | 105 | 309 | 1337 | 788 | 2125 | 0.561 | 0.52 | 1.08 | 1337.97 | 788.31 | 2126.28 |  | | | | |  |
| **Total** | **3499** | **1545** | **5044** | **53744** | **24608** | **78352** | **3** | **2** | **5** | **53747** | **24610** | **78357** | **2,700,374,100** | **1,021,113,322** | | **704,441,194** | **380,864,490** | |

| Table S21: Age-specific Health and Economic Burden Indicators Associated with COVID-19-Uttar Pradesh | | | | | | | | | | |  |  |  |  |  |  | |
| --- | --- | --- | --- | --- | --- | --- | --- | --- | --- | --- | --- | --- | --- | --- | --- | --- | --- |
| **Age group** | **Deaths** | | | **YLL discounted** | | | | **YLD** | | **DALYS** | | | **CPL due to premature mortality** | **CPL due to absenteeism from work** | **CPL due to premature mortality** | **CPL due to absenteeism from work** | |
|  | **Male** | **Female** | **Both** | **Male** | **Female** | **Both** | **Male** | **Female** | **Both** | **Male** | **Female** | **Both** | **Male** | | **Female** | |  |
| **<1** | 50 | 41 | 91 | 1431 | 1183 | 2615 | 0.036 | 0.03 | 0.06 | 1431.50 | 1183.45 | 2614.95 |  |  |  |  | |
| **1-5** | 6 | 0 | 6 | 161 | 0 | 161 | 0.314 | 0.28 | 0.59 | 161.61 | 0.28 | 161.89 |  |  |  |  | |
| **6-10** | 14 | 7 | 21 | 396 | 215 | 611 | 0.375 | 0.34 | 0.71 | 396.62 | 215.40 | 612.02 |  |  |  |  | |
| **11-15** | 11 | 18 | 29 | 316 | 505 | 821 | 0.712 | 0.61 | 1.32 | 316.59 | 505.89 | 822.48 |  |  |  |  | |
| **16-20** | 68 | 40 | 108 | 1835 | 1080 | 2915 | 1.210 | 0.95 | 2.16 | 1836.60 | 1080.72 | 2917.32 | 8,951,027 | 76,935,372 | 1,475,973 | 13,115,344 | |
| **21-25** | 113 | 113 | 226 | 2940 | 2984 | 5924 | 2.375 | 1.79 | 4.16 | 2942.11 | 2985.85 | 5927.96 | 174,607,678 | 854,215,306 | 20,163,612 | 85,686,077 | |
| **26-30** | 359 | 202 | 562 | 8906 | 5134 | 14040 | 3.548 | 2.56 | 6.11 | 8909.40 | 5136.86 | 14046.26 |  |  |  |  |  |
| **31-35** | 569 | 259 | 828 | 13372 | 6266 | 19638 | 3.489 | 2.28 | 5.76 | 13375.80 | 6268.05 | 19643.84 | 819,123,777 | 1,123,100,004 | 115,790,252 | 203,952,125 | |
| **35-40** | 982 | 442 | 1424 | 21693 | 10081 | 31773 | 3.620 | 2.33 | 5.95 | 21696.52 | 10082.93 | 31779.44 |  |  |  |  |  |
| **40-45** | 1266 | 685 | 1951 | 25944 | 14585 | 40529 | 3.564 | 2.23 | 5.80 | 25947.84 | 14587.02 | 40534.86 | 1,336,235,631 | 850,529,315 | 272,611,904 | 216,463,656 | |
| **45-50** | 1366 | 720 | 2086 | 25637 | 14090 | 39727 | 3.225 | 2.18 | 5.40 | 25640.65 | 14091.80 | 39732.44 |  |  |  |  |  |
| **50-55** | 1641 | 949 | 2590 | 27595 | 16776 | 44372 | 3.135 | 2.15 | 5.29 | 27598.49 | 16778.44 | 44376.93 | 969,678,702 | 640,904,046 | 224,145,081 | 186,737,721 | |
| **55-60** | 1836 | 1174 | 3010 | 27103 | 18540 | 45643 | 2.980 | 2.12 | 5.10 | 27105.71 | 18542.43 | 45648.14 |  |  |  |  |  |
| **60-65** | 2328 | 1355 | 3683 | 30157 | 18688 | 48845 | 2.351 | 1.65 | 4.00 | 30159.68 | 18689.78 | 48849.45 |  |  |  |  | |
| **65-70** | 2006 | 725 | 2732 | 22000 | 8483 | 30483 | 1.532 | 1.03 | 2.57 | 22001.61 | 8483.99 | 30485.60 |  |  |  |  | |
| **70-75** | 1181 | 471 | 1652 | 10639 | 4515 | 15154 | 0.912 | 0.59 | 1.50 | 10639.72 | 4515.95 | 15155.67 |  |  |  |  | |
| **75-80** | 830 | 342 | 1172 | 6032 | 2618 | 8650 | 0.586 | 0.36 | 0.95 | 6032.21 | 2618.31 | 8650.52 |  | | | |  |
| **80+** | 537 | 183 | 720 | 2858 | 992 | 3850 | 0.561 | 0.52 | 1.08 | 2858.68 | 992.51 | 3851.19 |  | | | |  |
| **Total** | **15164** | **7727** | **22891** | **229017** | **126736** | **355752** | **35** | **24** | **59** | **229051** | **126760** | **355811** | **3,308,596,815** | **3,545,684,044** | **634,186,822** | **705,954,924** | |

| Table S22: Age-specific Health and Economic Burden Indicators Associated with COVID-19-Uttarakhand | | | | | | | | | | | | |  |  |  |  |
| --- | --- | --- | --- | --- | --- | --- | --- | --- | --- | --- | --- | --- | --- | --- | --- | --- |
| **Age group** | **Deaths** | | | **YLL discounted** | | | | **YLD** | | **DALYS** | | | **CPL due to premature mortality** | **CPL due to absenteeism from work** | **CPL due to premature mortality** | **CPL due to absenteeism from work** |
|  | **Male** | **Female** | **Both** | **Male** | **Female** | **Both** | **Male** | **Female** | **Both** | **Male** | **Female** | **Both** | **Male** | | **Female** | |
| **<1** | 0 | 0 | 0 | 0 | 0 | 0 | 0.037 | 0.02 | 0.06 | 0.04 | 0.02 | 0.06 |  |  |  |  |
| **1-5** | 0 | 0 | 0 | 0 | 0 | 0 | 0.040 | 0.04 | 0.08 | 0.04 | 0.04 | 0.08 |  |  |  |  |
| **6-10** | 0 | 0 | 0 | 0 | 0 | 0 | 0.055 | 0.05 | 0.10 | 0.05 | 0.05 | 0.10 |  |  |  |  |
| **11-15** | 13 | 11 | 24 | 361 | 316 | 677 | 0.137 | 0.12 | 0.26 | 360.98 | 316.32 | 677.29 |  |  |  |  |
| **16-20** | 17 | 19 | 36 | 457 | 529 | 985 | 0.253 | 0.21 | 0.46 | 456.75 | 529.08 | 985.84 | 3,542,931 | 29,067,438 | 836,024 | 10,894,714 |
| **21-25** | 53 | 40 | 92 | 1368 | 1078 | 2446 | 0.451 | 0.37 | 0.82 | 1368.00 | 1078.49 | 2446.49 | 120,532,922 | 435,536,974 | 33,754,431 | 184,435,844 |
| **26-30** | 77 | 60 | 138 | 1915 | 1587 | 3502 | 0.633 | 0.49 | 1.12 | 1916.03 | 1587.39 | 3503.42 |  |  |  |  |
| **31-35** | 153 | 87 | 240 | 3586 | 2189 | 5775 | 0.654 | 0.45 | 1.10 | 3586.61 | 2189.03 | 5775.63 | 581,453,960 | 746,069,872 | 152,471,947 | 291,211,188 |
| **35-40** | 213 | 111 | 324 | 4713 | 2670 | 7384 | 0.683 | 0.46 | 1.14 | 4713.94 | 2670.80 | 7384.74 |  |  |  |  |
| **40-45** | 343 | 130 | 473 | 7048 | 2943 | 9991 | 0.671 | 0.44 | 1.11 | 7048.55 | 2943.42 | 9991.97 | 1,129,118,994 | 549,330,065 | 192,802,245 | 230,247,809 |
| **45-50** | 396 | 196 | 592 | 7469 | 4124 | 11593 | 0.563 | 0.42 | 0.98 | 7469.10 | 4124.85 | 11593.95 |  |  |  |  |
| **50-55** | 494 | 243 | 738 | 8454 | 4703 | 13157 | 0.535 | 0.39 | 0.93 | 8454.59 | 4703.72 | 13158.31 | 877,284,486 | 369,538,636 | 201,654,260 | 187,203,368 |
| **55-60** | 592 | 294 | 887 | 8972 | 5106 | 14078 | 0.494 | 0.38 | 0.88 | 8972.16 | 5106.44 | 14078.60 |  |  |  |  |
| **60-65** | 643 | 298 | 941 | 8526 | 4612 | 13138 | 0.383 | 0.29 | 0.67 | 8526.42 | 4612.51 | 13138.93 |  |  |  |  |
| **65-70** | 632 | 277 | 909 | 7014 | 3676 | 10690 | 0.257 | 0.19 | 0.45 | 7014.10 | 3676.55 | 10690.65 |  |  |  |  |
| **70-75** | 513 | 279 | 792 | 5062 | 3228 | 8290 | 0.166 | 0.12 | 0.29 | 5062.16 | 3228.54 | 8290.70 |  |  |  |  |
| **75-80** | 396 | 202 | 598 | 3214 | 1963 | 5178 | 0.110 | 0.08 | 0.19 | 3214.39 | 1963.36 | 5177.76 |  | | | |
| **80+** | 447 | 162 | 609 | 3491 | 1518 | 5009 | 0.561 | 0.52 | 1.08 | 3492.03 | 1518.33 | 5010.36 |  | | | |
| **Total** | **4983** | **2410** | **7393** | **71649** | **40244** | **111893** | **7** | **5** | **12** | **71656** | **40249** | **111905** | **2,711,933,294** | **2,129,542,985** | **581,518,907** | **903,992,922** |

| Table S23: Age-specific Health and Economic Burden Indicators Associated with COVID-19-West Bengal | | | | | | | | | |  |  |  |  |  |  |  |
| --- | --- | --- | --- | --- | --- | --- | --- | --- | --- | --- | --- | --- | --- | --- | --- | --- |
| **Age group** | **Deaths** | | | **YLL discounted** | | | | **YLD** | | **DALYS** | | | **CPL due to premature mortality** | **CPL due to absenteeism from work** | **CPL due to premature mortality** | **CPL due to absenteeism from work** |
|  | **Male** | **Female** | **Both** | **Male** | **Female** | **Both** | **Male** | **Female** | **Both** | **Male** | **Female** | **Both** | **Male** |  | **Female** |  |
| 0-15 | 33 | 23 | 56 | 968 | 680 | 1647 | 0.889 | 0.860 | 1.749 | 968.42 | 680.62 | 1649.04 |  |  |  |  |
| 16-30 | 201 | 118 | 320 | 5343 | 3211 | 8554 | 3.260 | 3.168 | 6.428 | 5346.41 | 3213.83 | 8560.24 | 120793150.4 | 1133290223 | 15827252 | 45005533.66 |
| 31-45 | 996 | 490 | 1486 | 22997 | 11666 | 34663 | 4.583 | 4.197 | 8.780 | 23001.75 | 11670.35 | 34672.10 | 1033257615 | 2480074621 | 151931657.6 | 423265593.5 |
| 46-60 | 3439 | 1545 | 4984 | 62033 | 29157 | 91190 | 4.257 | 3.987 | 8.243 | 62037.18 | 29160.91 | 91198.09 | 3546481598 | 2055959508 | 377012466.7 | 403361708.7 |
| 61-75 | 5242 | 2582 | 7824 | 62762 | 32916 | 95678 | 2.489 | 2.183 | 4.672 | 62764.45 | 32918.15 | 95682.60 |  |  |  |  |
| 75+ | 2690 | 1448 | 4138 | 18278 | 9842 | 28120 | 0.623 | 0.510 | 1.134 | 18278.38 | 9842.38 | 28120.76 |  |  |  |  |
| **Total** | **12601** | **6207** | **18808** | **172380** | **87471** | **259852** | **16** | **15** | **31** | **172397** | **87486** | **259883** | **4,700,532,364** | **5,669,324,352** | **544,771,376** | **871,632,836** |

| Table S24: Age-specific Health and Economic Burden Indicators Associated with COVID-19-Other Union territories | | | | | | | | | | | | | |  | |  |  |
| --- | --- | --- | --- | --- | --- | --- | --- | --- | --- | --- | --- | --- | --- | --- | --- | --- | --- |
| **Age group** | **Deaths** | | | **YLL discounted** | | | | **YLD** | | **DALYS** | | | **CPL due to premature mortality** | **CPL due to absenteeism from work** | | **CPL due to premature mortality** | **CPL due to absenteeism from work** |
|  | **Male** | **Female** | **Both** | **Male** | **Female** | **Both** | **Male** | **Female** | **Both** | **Male** | **Female** | **Both** | **Male** | | **Female** | | |
| **<1** | 1 | 1 | 2 | 30 | 24 | 54 | 0.065 | 0.01 | 0.07 | 30.05 | 24.09 | 54.14 |  |  | |  |  |
| **1-9** | 3 | 2 | 5 | 85 | 48 | 133 | 0.194 | 0.12 | 0.31 | 84.77 | 48.29 | 133.06 |  | | | | |
| **10-19** | 6 | 5 | 12 | 178 | 153 | 331 | 0.380 | 0.27 | 0.65 | 177.90 | 153.53 | 331.43 | 1,228,205 | 39,911,247 | | 1,232,653 | 23,001,892 |
| **20-29** | 38 | 23 | 61 | 996 | 627 | 1623 | 0.723 | 0.61 | 1.33 | 996.89 | 627.32 | 1624.21 | 194,325,334 | 1,528,430,895 | | 111,048,929 | 958,502,558 |
| **30-39** | 131 | 51 | 182 | 3147 | 1277 | 4423 | 0.850 | 0.71 | 1.56 | 3147.60 | 1277.24 | 4424.83 | 916,309,559 | 1,809,169,316 | | 462,555,104 | 1,645,638,625 |
| **40-49** | 256 | 120 | 376 | 5410 | 2641 | 8051 | 0.821 | 0.73 | 1.55 | 5410.90 | 2641.74 | 8052.64 | 1,862,194,129 | 1,504,438,629 | | 776,449,227 | 1,105,878,692 |
| **50-59** | 418 | 222 | 641 | 7355 | 4135 | 11490 | 0.632 | 0.62 | 1.25 | 7355.76 | 4135.83 | 11491.59 | 1,121,433,789 | 659,982,210 | | 494,468,082 | 530,739,275 |
| **60-69** | 539 | 302 | 840 | 7332 | 4406 | 11738 | 0.340 | 0.37 | 0.71 | 7332.66 | 4406.17 | 11738.82 |  |  | |  |  |
| **70-79** | 420 | 211 | 632 | 4058 | 2188 | 6245 | 0.142 | 0.15 | 0.29 | 4057.75 | 2187.95 | 6245.70 |  | | | | |
| **80+** | 201 | 99 | 300 | 1237 | 642 | 1879 | 0.047 | 0.05 | 0.10 | 1237.12 | 641.96 | 1879.08 |  | | | | |
| **Total** | **2013** | **1037** | **3050** | **29827** | **16140** | **45968** | **4** | **4** | **8** | **29831** | **16144** | **45976** | **4,095,491,016** | **5,541,932,298** | | **1,845,753,995** | **4,263,761,042** |

| Table S25: Age-specific Health and Economic Burden Indicators Associated with COVID-19-Other North-east states | | | | | | | | | | |  |  |  |  |  |  |
| --- | --- | --- | --- | --- | --- | --- | --- | --- | --- | --- | --- | --- | --- | --- | --- | --- |
| **Age group** | **Deaths** | | | **YLL discounted** | | | | **YLD** | | **DALYS** | | | **CPL due to premature mortality** | **CPL due to absenteeism from work** | **CPL due to premature mortality** | **CPL due to absenteeism from work** |
|  | **Male** | **Female** | **Both** | **Male** | **Female** | **Both** | **Male** | **Female** | **Both** | **Male** | **Female** | **Both** | **Male** |  | **Female** |  |
| <1 | 8 | 3 | 12 | 238 | 99 | 337 | 0.035 | 0.032 | 0.066 | 237.71 | 99.40 | 337.11 |  |  |  |  |
| 1-15 | 8 | 3 | 11 | 232 | 100 | 332 | 0.861 | 0.853 | 1.715 | 232.93 | 101.03 | 333.96 |  |  |  |  |
| 16-20 | 24 | 10 | 34 | 646 | 281 | 927 | 0.480 | 0.478 | 0.957 | 646.14 | 281.35 | 927.49 | 4580065.194 | 35248577.75 | 841236.4642 | 14924594.19 |
| 21-30 | 279 | 120 | 399 | 7265 | 3169 | 10434 | 1.423 | 1.306 | 2.730 | 7266.04 | 3170.52 | 10436.56 | 241725663 | 492657301.3 | 46112221 | 177863749.9 |
| 31-45 | 957 | 410 | 1367 | 22658 | 9963 | 32621 | 2.368 | 1.902 | 4.269 | 22660.07 | 9965.26 | 32625.34 | 1196425194 | 1016180809 | 256376827.2 | 345602964.8 |
| 46-60 | 1236 | 530 | 1766 | 23521 | 10479 | 33999 | 1.578 | 1.324 | 2.902 | 23522.11 | 10479.88 | 34001.99 | 1506747978 | 523605335.9 | 324944275.5 | 200321572.1 |
| 61-75 | 997 | 427 | 1424 | 13214 | 5942 | 19156 | 0.705 | 0.642 | 1.347 | 13214.96 | 5942.31 | 19157.26 |  |  |  |  |
| 75+ | 478 | 205 | 684 | 3664 | 1664 | 5328 | 0.199 | 0.172 | 0.371 | 3663.94 | 1664.27 | 5328.21 |  |  |  |  |
| **Total** | **3987** | **1709** | **5696** | **71436** | **31697** | **103134** | **8** | **7** | **14** | **71444** | **31704** | **103148** | **2,949,478,900** | **2,067,692,024** | **628,274,560** | **738,712,881** |
